# Polyclonal B Cell Differentiation and Loss of Gastrointestinal Tract Germinal Centers in the Earliest Stages of HIV-1 Infection

**DOI:** 10.1371/journal.pmed.1000107

**Published:** 2009-07-07

**Authors:** Marc C. Levesque, M. Anthony Moody, Kwan-Ki Hwang, Dawn J. Marshall, John F. Whitesides, Joshua D. Amos, Thaddeus C. Gurley, Sallie Allgood, Benjamin B. Haynes, Nathan A. Vandergrift, Steven Plonk, Daniel C. Parker, Myron S. Cohen, Georgia D. Tomaras, Paul A. Goepfert, George M. Shaw, Jörn E. Schmitz, Joseph J. Eron, Nicholas J. Shaheen, Charles B. Hicks, Hua-Xin Liao, Martin Markowitz, Garnett Kelsoe, David M. Margolis, Barton F. Haynes

**Affiliations:** 1 Department of Medicine, Duke University School of Medicine, Durham, North Carolina, United States of America; 2 Department of Immunology, Duke University School of Medicine, Durham, North Carolina, United States of America; 3 The Duke Human Vaccine Institute, Duke University School of Medicine, Durham, North Carolina, United States of America; 4 Department of Pediatrics, Duke University School of Medicine, Durham, North Carolina, United States of America; 5 Department of Medicine, University of North Carolina at Chapel Hill, North Carolina, United States of America; 6 Department of Surgery, Duke University School of Medicine, Durham, North Carolina, United States of America; 7 Department of Medicine, University of Alabama Birmingham, Birmingham, Alabama, United States of America; 8 Division of Viral Pathogenesis, Beth Israel Deaconess Medical Center, Harvard Medical School, Boston, Massachusetts, United States of America; 9 Aaron Diamond AIDS Research Center, New York, New York, United States of America; National Institutes of Health, United States of America

## Abstract

**Background:**

The antibody response to HIV-1 does not appear in the plasma until approximately 2–5 weeks after transmission, and neutralizing antibodies to autologous HIV-1 generally do not become detectable until 12 weeks or more after transmission. Moreover, levels of HIV-1–specific antibodies decline on antiretroviral treatment. The mechanisms of this delay in the appearance of anti-HIV-1 antibodies and of their subsequent rapid decline are not known. While the effect of HIV-1 on depletion of gut CD4^+^ T cells in acute HIV-1 infection is well described, we studied blood and tissue B cells soon after infection to determine the effect of early HIV-1 on these cells.

**Methods and Findings:**

In human participants, we analyzed B cells in blood as early as 17 days after HIV-1 infection, and in terminal ileum inductive and effector microenvironments beginning at 47 days after infection. We found that HIV-1 infection rapidly induced polyclonal activation and terminal differentiation of B cells in blood and in gut-associated lymphoid tissue (GALT) B cells. The specificities of antibodies produced by GALT memory B cells in acute HIV-1 infection (AHI) included not only HIV-1–specific antibodies, but also influenza-specific and autoreactive antibodies, indicating very early onset of HIV-1–induced polyclonal B cell activation. Follicular damage or germinal center loss in terminal ileum Peyer's patches was seen with 88% of follicles exhibiting B or T cell apoptosis and follicular lysis.

**Conclusions:**

Early induction of polyclonal B cell differentiation, coupled with follicular damage and germinal center loss soon after HIV-1 infection, may explain both the high rate of decline in HIV-1–induced antibody responses and the delay in plasma antibody responses to HIV-1.

*Please see later in the article for Editors' Summary*

## Abstract 2

Studying the effects of early HIV infection on human antibody responses, M. Anthony Moody and colleagues find rapid polyclonal B cell differentiation and structural damage to gut-associated lymphoid tissue.

## Editors' Summary

**Background:**

Acquired immunodeficiency syndrome (AIDS) has killed more than 25 million people since 1981 and more than 30 million people are now infected with the human immunodeficiency virus (HIV), which causes AIDS. HIV infects and kills a type of immune system cell called CD4^+^ T lymphocytes. These cells are needed to maintain a vigorous immune response, so people infected with HIV eventually become susceptible to other infections and develop full-blown AIDS. However, early during HIV infection, other parts of the immune system attempt to fight off the virus. Soon after infection, immune system cells called B lymphocytes begin to produce HIV-specific antibodies (proteins that recognize viral molecules called antigens). The first antibodies to HIV usually appear two to seven weeks after infection; from about 12 weeks after infection, antibodies are made that can kill the specific HIV type responsible for the infection (neutralizing antibodies).

**Why Was This Study Done?:**

Unfortunately, by this time, it is too late for the antibody (“humoral”) immune response to clear HIV from the body. Indeed, the humoral immune response to HIV is very slow; for most viruses, neutralizing antibodies appear within days of infection. To help them design an effective HIV vaccine, scientists need to understand how the virus delays humoral responses to HIV infection (and how it later causes the production of HIV-specific antibodies to decline). Little is known, however, about the early effects of HIV infection on B lymphocytes. These cells are born and mature in the bone marrow. “Naïve” B lymphocytes, each of which carries an antigen-specific receptor (a protein that binds to a specific antigen), then enter the blood and circulate around the body, passing through the “peripheral lymphoid organs”. Exposure to antigens in these organs, which include lymph nodes and gut-associated lymphoid tissues, activates the subset of B lymphocytes that recognize the specific antigens that are present. Finally, with the help of activated T lymphocytes, the activated B lymphocytes proliferate and change (differentiate) into antibody-secreting cells and memory B lymphocytes (which respond more quickly to antigen than naïve B lymphocytes). In this study, the researchers investigate the effects of early HIV-1 infection on B lymphocytes in blood and in gut-associated lymphoid tissues.

**What Did the Researchers Do and Find?:**

The researchers collected blood from patients as early as 17 days after HIV-1 infection and tissue samples from the lower portion of the small intestine (a region rich in gut-associated lymphoid structures called Peyer's patches) from 47 days after infection onward. When they analyzed the B lymphocytes in these samples (which were collected during two trials organized by the US Center for HIV/AIDS Vaccine Immunology [CHAVI]), they found that HIV-1 infection rapidly induced the activation of many different B cells that recognized a variety of antigens (polyclonal activation), as well as the appearance of differentiated B cells in blood and in gut-associated lymphoid tissue. The B lymphocytes that were activated in the gut made HIV-specific antibodies but also antibodies against unrelated antigens (such as flu virus proteins). Finally, the structure of Peyer's patches was altered early in HIV-1 infection. More specifically, most of the lymphoid follicles (organized collections of lymphocytes and antigen-presenting cells) in the Peyer's patches showed signs of damage and T- and B-lymphocyte death and the number of germinal centers (regions in lymphoid follicles in which B lymphocytes proliferate) was reduced.

**What Do These Findings Mean?:**

Although the depletion of gut-associated CD4+ T lymphocytes in early HIV-1 infection is well known, these new results demonstrate the effects of early HIV-1 infection on gut-associated and circulating B lymphocytes. The results of this study are limited by the methods used to analyze the antibodies induced by HIV infection and by only taking tissue samples from one region of the gut. Nevertheless, the findings of polyclonal B-cell activation and damage to gut-associated lymphoid follicles soon after HIV-1 infection may have implications for HIV-1 vaccine design. Specifically, these findings suggest that an effective HIV-1 vaccine will need to ensure that significant levels of neutralizing antibodies are present in people before HIV-1 infection and that other protective immune defenses are fully primed so that, in the event of HIV-1 infection, the virus can be dealt with effectively before it disables any part of the immune system.

**Additional Information:**

Please access these Web sites via the online version of this summary at http://dx.doi.org/10.1371/journal.pmed.1000107.

- Information is available from the US National Institute of Allergy and Infectious Diseases on HIV infection and AIDS
- HIV InSite has comprehensive information on all aspects of HIV/AIDS, including an article about how HIV-1 infection affects the immune system
- Information is available from Avert, an international AIDS charity on many aspects of HIV/AIDS, including information on the stages of HIV infection, and on AIDS vaccines (in English and Spanish)
- The US Center for HIV/AIDS Vaccine Immunology (CHAVI) Web site provides information on research designed to solve major problems in HIV vaccine development and design

## Introduction

Early HIV-1 infection is characterized by the death of both infected and uninfected CD4^+^ T cells, often resulting in extensive CD4^+^ T cell depletion in the gastrointestinal tract [1]–[6]. While HIV-1 induction of CD4^+^ T cell death in early infection is well documented, the effects of HIV-1 on blood and mucosal B cells and their inductive microenvironments in the earliest stages of infection have not been determined. In chronic infection, HIV-1 induces polyclonal B-cell activation, B cell exhaustion, hypergammaglobulinemia, reductions in the numbers of memory B cells in the blood, and increased numbers of circulating immature B cells [7]–[12]. People with early HIV-1 infection have elevated frequencies of blood antibody-secreting cells that may reflect Env-mediated polyclonal activation [10],[13],[14], but have been reported to have normal numbers of blood naïve and memory B cells [15].

Priming immunizations of healthy humans with nonpathogenic non-HIV-1 viruses elicit detectable neutralizing antibody within a few days of priming, with peak neutralization titers observed on day 14 [16],[17]. In contrast, the earliest B cell responses to HIV-1 are not detected until ∼19 d after transmission in the form of antibody–virion immune complexes, and ∼2–5 wk after transmission in the form of HIV-1 antibodies in plasma [18]. Most importantly, functionally relevant neutralizing antibodies against autologous virus do not generally appear in plasma until ≥12 wk after HIV-1 infection [19]–[21]. Moreover, antiretroviral therapy in chronic HIV-1 infection is associated with an increased rate of decline of plasma antibodies resulting in an apparent half-life of anti-HIV-1 gp120 antibody of 7–21 wk, and for anti-p24 antibody of 9–15 wk [14]. The mechanisms of delay in the appearance of anti-HIV-1 antibodies after HIV-1 transmission and of the rapid decline of induced anti-HIV-1 antibodies are not known.

Given the potential capacity of neutralizing serum antibody to prevent HIV-1 infection, as demonstrated by passive transfer of protective antibody to rhesus macaques challenged with simian human immunodeficiency virus [22],[23], it is critical to define the early effects of HIV-1 infection on B cells and on their inductive and effector mucosal microenvironments. Detailed examination of how HIV-1 subverts initial humoral responses will determine how quickly vaccine-primed neutralizing antibodies must appear in order to be effective. In this study, we investigated the effects of acute and early HIV-1 infection on naïve B cells, memory B cells, and plasma cells in blood, Peyer's patches, and lamina propria.

## Methods

### Ethics Statement

This study was conducted according to the principles expressed in the Declaration of Helsinki. The study was approved by the Institutional Review Board of Duke University Medical Center, the University of North Carolina Medical Center, the University of Alabama Birmingham, and the Aaron Diamond AIDS Research Center. All patients provided written informed consent for the collection of samples and subsequent analysis.

### Study Participants

Patients were recruited for terminal ileum (Table 1) and blood samples (Table 2) under IRB-approved protocols for CHAVI 001 and CHAVI 012 studies of acute HIV-1 infection. Control samples of blood were obtained from healthy uninfected volunteers; control samples for terminal ileum biopsies were obtained from healthy uninfected volunteers or as de-identified and discarded tissue samples from the Duke Surgical Pathology Department from patients undergoing surgery for colon cancer, colon polyps, or endometriosis. Differences between HIV-1 infected patients who were on or off antiretroviral treatment (ART) were not statistically significant (*t*-test) for age, time from transmission, or cell counts (CD3^+^, CD4^+^, CD8^+^) for both those contributing terminal ileum biopsies and those contributing peripheral blood (Tables 1 and 2). For viral load (VL) data, tests were performed on the natural logarithm of VL and were not statistically significant (*t*-test) between the on- and off-ART groups except for plasma VL for participants contributing peripheral blood (*p* = 0.035, *t*-test). Time from transmission was precisely known in many patients by history, and was inferred clinically in others from their last high-risk behavior prior to documented infection. Many participants came from the CHAVI 001 acute HIV-1 infection protocol that detects plasma VL-positive, serum antibody–negative AHI patients by VL screening. The patients studied were classified as having either early acute HIV-1 infection (≤40 d after transmission) or early HIV infection (41–200 d after transmission) (Tables 1 and 2). In this study all patients were within the first 200 d of infection and are analyzed together as “acute,” although those who donated after day 40 from transmission are only early in infection. In the CHAVI 012 protocol, participants were asked to donate terminal ileum biopsies and paired blood samples. Six of the 14 patients who donated terminal ileum samples elected not to donate paired blood samples and thus were not included in the blood B cell analysis presented in Table 2. There was no obvious source of selection bias noted for the groups in Tables 1 and 2.

**Table 1:** Participants who contributed terminal ileum tissues.

Participants^a^	Sex	ART^b^	Age (y)	Time at Tissue Biopsy (d)		Viral Load (copies/ml)	GALT HIV-1 RNA (copies/µg)	CD3 (cells/µl)	CD4 (cells/µl)		CD8 (cells/µl)
				From Transmission	From Initiation of ART				At Biopsy	At Day 168	
**AHI off ART**											
003-7	M	No	53	114	N/A	91,713	526	1,560	540	720	960
011-6	M	No	18	95	N/A	27,869	44,816	2,336	522	554	1,621
017-9	M	No	26	56	N/A	6,570	106	1,478	701	487	758
*019-2*	M	No	18	47	N/A	24,545	83	2,310	973	741	1,216
020-4	M	No	19	113	N/A	57,689	38	1,088	414	379	628
*023-3*	M	No	17	66	N/A	203	72	NR	1,032	901	704
**Mean**	—	—	25.2	82	N/A	13,000^c^	288^c^	1,754	697	630	981
**AHI on ART**											
004-5	M	Yes	41	55	21	13,015	254	1,718	673	561	998
*008-6*	M	Yes	26	67	17	464	10,639	1,369	582	775	732
*010-1*	M	Yes	19	52	14	887	4,072	2,032	940	789	1,041
015-5	M	Yes	28	55	14	123,928	338	2,109	801	898	1,308
*016-8*	M	Yes	40	147	32	387,344	574	3,468	478	938	2,870
*018-4*	M	Yes	25	74	29	1,513	<50	1,220	599	621	556
*025-7*	F	Yes	30	105	63	1,225	<50	1,946	691	804	1,101
*016-2*	M	Yes	28	80	59	<50	NR	1,362	583	659	NR
**Mean**	—	—	29.6	79	31	3,525^c^	474^c^	1,903	668	756	1,229
**Overall Mean**	—	—	27.7	80	—	6,167^c^	376^c^	1,846	681	702	1,115

Tissues from nine uninfected participants were obtained as terminal ileal biopsies from healthy volunteers or as discarded specimens from surgeries for colon cancer, colon polyps, carcinoid tumor, and endometriosis.

a Participants with GALT germinal centers indicated by italics.

b All participants herein received efavirenz, tenofovir, and emtricitibine daily.

c Viral load comparisons reported as geometric means.

N/A, not applicable; NR, not reported.

**Table 2:** Participants who contributed peripheral blood.

Participants	Sex	ART^a^	Age (y)	Time from Transmission (d)	Time from Initiation of ART (d)	Viral Load (copies/ml)	CD3 (cells/µl)	CD4 (cells/µl)	CD8 (cells/µl)
**AHI off ART**									
003-7	M	No	53	129	N/A	45,002	1,238	398	780
013-0	M	No	24	99	N/A	<400	1,515	708	768
019-2	M	No	18	38	N/A	24,545	2,310	973	1,216
008-0	M	No	21	165	N/A	5,646	2,233	924	NR
012-1	M	No	24	184	N/A	2,878	1,385	644	NR
054-4	M	No	41	40	N/A	88,900	1,160	434	732
055-9	M	No	24	49	N/A	3,380,000	1,781	411	1,338
056-7	M	No	36	83	N/A	3,090	1,239	455	699
058-0	M	No	53	46	N/A	3,560,000	1,887	393	1,608
063-5	M	No	42	39	N/A	525,000	4,073	503	3,543
065-0	M	No	35	17	N/A	1,860,000	694	269	397
008-1	M	No	45	36	N/A	3,746	1,840	805	989
024-6	M	No	25	21	N/A	4,395,721	2,206	1,012	1,142
025-2	M	No	17	33	N/A	12,803	1,669	722	857
6846	M	No	32	20	N/A	15,300,000	480	225	218
068-9	M	No	37	30	N/A	1,280	1,140	611	518
6817	M	No	42	20	N/A	9,000,000	NR	230	NR
**Mean**	—	—	33.5	62	N/A	86,568^b^	1,678	572	1,057
**AHI on ART**									
004-5	M	Yes	41	91	57	13,015	1,718	673	998
006-1	M	Yes	25	192	170	<50	1,665	851	776
008-6	M	Yes	26	76	26	464	1,369	582	732
010-1	M	Yes	19	67	29	336	2,032	940	1,041
014-3	M	Yes	32	77	24	7,417	1,234	484	701
015-5	M	Yes	28	54	11	123,928	2,109	801	1,308
016-8	M	Yes	40	127	12	387,344	3,468	478	2,870
018-4	M	Yes	25	66	21	2,921	1,220	599	556
021-8	M	Yes	24	64	29	12,574	1,258	561	677
**Mean**	—	—	28.9	90	42	8,472^b^	1,786	663	1,073
**Overall Mean**	—	—	31.9	72	—	31,785^b^	1,717	603	1,064

There were 14 uninfected control participants who contributed peripheral blood (36% male, mean age 33.5 y [range18–66 y]).

a All participants herein received efavirenz, tenofovir, and emtricitibine daily.

b Viral load comparisons reported as geometric means and were different between on and off ART groups (*p* = 0.035, *t*-test).

N/A, not applicable; NR, not reported.

### Flow Cytometry Analysis of Terminal Ileum and Blood B Cells

Terminal ileum mononuclear cells were isolated from gut tissues followed by Ficoll-Hypaque density gradient centrifugation and were not further enriched prior to antibody staining. Peripheral blood B cells were enriched before antibody staining using RosetteSep B-cell enrichment cocktail (STEMCELL Technologies). A panel of monoclonal antibodies was used to identify distinct human B cell subsets in various tissues using nine-color flow cytometry on a BD FACSAria (BD Biosciences). B cells were defined as CD3^−^/CD14^−^/CD16^−^/CD235a^−^ and CD19^+^. B cell subsets were defined as follows: naïve B cells as IgD^+^, IgM^+^, CD27^−^; memory B cells as IgD^−^, CD38^−/+^, CD27^+^; plasmablasts/plasma cells as IgD^−^, CD38^hi^, CD27^+^ (Text S1) [24],[25]. Combinations of the following antibodies were used: IgM (FITC), IgD (PE), CD3 (PE-Cy5), CD4 (PE-Cy7), CD5 (PE-Cy7), CD8 (APC), CD14 (PE, PE-Cy5), CD16 (FITC, PE-Cy5), CD19 (APC-Cy7), CD20 (PE-Cy7), CD27 (PacificBlue), CD56 (PE-Cy5), CD235a (PE-Cy5) from BD Biosciences; CD3 (APC-Cy5.5), CD14 (PE-Cy5), CD38 (APC-Cy5.5 or AlexaFluor 700), CD45 (PacificBlue) from Caltag/Invitrogen; CD38 (APC-Cy5.5 or AlexaFluor 700) from eBioscience.

### Immunohistologic and Quantitative Image Analysis of Terminal Ileum Tissue Immune Cells

Formalin-fixed biopsies of terminal ileum were cut into 5 µm thick sections, treated for antigen retrieval when required using standard techniques [26], and stained with saturating amounts of the monoclonal or polyclonal antibodies against: CD3, CD4, CD8, CD57, CD16, Langerin, CD83, CD11c, CD56, CD68, CD20, IgG, IgM, IgA, kappa and lambda light chains, CD21, CD35, and nerve growth factor receptor p75. Secondary stains were with horseradish peroxidase-labeled polyclonal IgG (H+L) against the species of the primary antibody followed by development with 3,3-diaminobenzidine in the presence of hydrogen peroxide. Some slides were prepared with TUNEL stains for apoptotic cells using standard techniques (see Table S1 for antibody sources).

For quantitative image analysis, the area in cubic millimeters of a 40× field was evaluated using an LCD monitor and a Nikon D5-Fi1 camera with the NIS Elements F3 program. Five 40× fields were photographed and the average number of lamina propria cells per field that stained positive for the monoclonal antibody were counted [27]. The number of cells/mm^3^ of tissue was calculated by the equation:
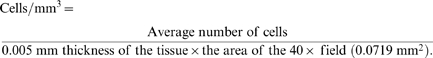



Peyer's patch germinal centers were identified by a combination of morphology in hematoxylin and eosin stains, the presence of Ki-67^+^ dividing germinal center cells, and identification of follicular dendritic cells (FDCs). FDCs were identified by reactivity with CD21, CD35, or anti-nerve growth factor receptor p75 monoclonal antibodies [23], and similar results were found using each reagent. Follicular damage and loss of germinal centers (follicular lysis) was determined by TUNEL stains for apoptotic cells in T and B cell areas, CD8^+^ T cell and macrophage infiltration of follicles, visible damage to follicles visible by hematoxylin and eosin staining, loss of Ki-67^+^ (dividing) germinal center cells, and loss of the normal fine reticular pattern of FDCs. In some sections apoptotic bodies outside of tingible body macrophages could be seen within germinal centers on hematoxylin and eosin-stained sections.

### Epstein-Barr Virus B Cell Transformation and Assays for Antibody Specificity

Mucosal cells isolated from terminal ileal samples of participants were transformed with Epstein-Barr virus (EBV) to generate B-lymphoblastoid cell lines according to the methods described previously [28]. Briefly, the cells were resuspended in culture medium (RPMI supplemented with 15% heat-inactivated fetal calf serum, 100 units/ml penicillin G, 100 µg/ml streptomycin, and 50 µg/ml of gentamicin) and incubated with an EBV suspension (from B95-8 B cell line) in the presence of phytohemagglutinin (2 µg/ml; Sigma). After 4 h, cells were plated at 10^4^ cells per well in 96-well plates (round bottom).

Three to four weeks after plating, levels of total IgA, IgG, and IgM in the supernatants were determined by ELISA. Briefly, 96-well plates (Costar) were coated with 5 µg/ml of goat anti-human immunoglobulins (Invitrogen) in 0.1 M sodium bicarbonate buffer. After incubating overnight at 4°C, plates were blocked with PBS containing 15% goat serum, 4% whey protein, and 0.5% Tween-20. The supernatants (1∶25–1∶100 dilutions) were distributed to wells and incubated for 1 h at room temperature. In each plate, affinity-purified human IgA, IgG, or IgM in the range of 0–200 ng/ml (Jackson ImmunoResearch Laboratories) was included for standard curves. After washing with PBS containing 0.1% Tween-20, bound human IgA, IgG, and IgM were detected separately with isotype-specific, horseradish peroxidase-labeled goat anti-human immunoglobulins (Jackson ImmunoResearch Laboratories) and the peroxidase substrate tetramethylbenzidine (Kirkegaard and Perry Laboratories).

### Tissue and Plasma Viral Load Determinations

Plasma HIV-1 RNA was measured by Roche Amplicor assay. GALT samples obtained in the terminal ileum were flash frozen in liquid nitrogen followed by pulverization and nucleic acid extraction using the AllPrep DNA/RNA Mini Kit (Qiagen), as described [29]. Sample RNA was treated with Turbo DNase (Applied Biosystems) and proteinase K, extracted with phenol/chloroform and precipitated with ethanol prior to generating cDNA with the High-Capacity cDNA Reverse Transcription Kit (Applied Biosystems). HIV copy number was determined by quantitative PCR using a TaqMan probe labeled with FAM dye (IDT) to a conserved region of the HIV *gag* gene against a serial dilution of HIV *gag* cDNA synthesized from RNA transcribed from the p5′ plasmid (NIH AIDS Research and Reference Reagent Program, catalog #3119) as described [30]. The limit of detection for HIV-1 RNA is 20 copies/µg of total RNA. GAPD-DH was assessed in duplex as an input control using a TaqMan probe labeled with a CalFluor Orange 560 reporter (Biosearch Technologies). Sequences for all primers and probes were provided by S. Dandekar [29]. PCR products were analyzed with Sequence Detection Software Version 1.3.1 (Applied Biosystems).

### Plasma Antibody Levels

Plasma levels of IgM, IgG, and IgA antibodies specific for HIV-1 gp41, gp120, gp140, and p55, as well as levels of rheumatoid factor, were determined by ELISA as previously described [18],[31].

### Statistical Analysis

Statistical tests were performed in SAS v9.1 (SAS Institute). The comparisons for terminal ileum and peripheral blood were analyzed separately for each tissue type. In cases where there were more than two groups, multiple degree of freedom F-tests for groups were performed using PROC GLM in SAS v9.1, and subsequent pairwise comparisons were made. For data consisting of two groups only, *t*-tests using the Satterthwaite correction, two-tailed Fisher's exact tests, or two-tailed exact Wilcoxon tests were performed using the appropriate SAS PROC test in SAS v9.1; the statistical test used is noted when *p*-values are presented. Graphs of the data were created using GraphPad Prism (GraphPad Software).

## Results

### Effect of HIV-1 Infection on B Cells in Lamina Propria and Peyer's Patches of Terminal Ileum

**Figure 1:**
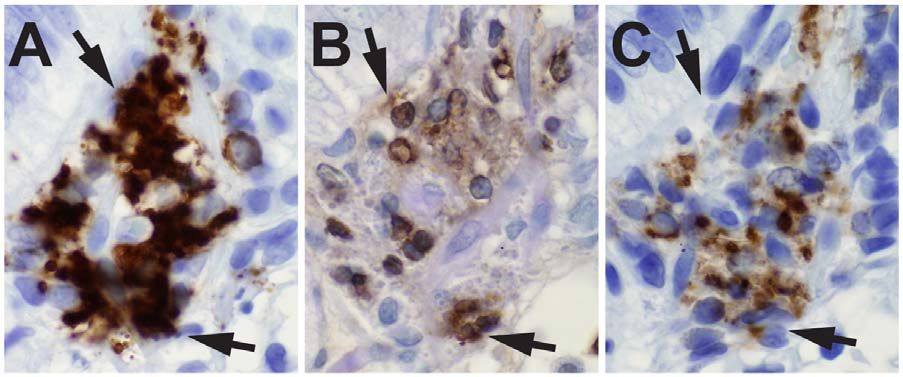
Effect of HIV-1 on B cells in terminal ileum lamina propria. Lamina propria B cell apoptosis with CD11c^+^ macrophage infiltration in participant 015-5, 55 d after transmission, on ART 15 d. A cluster of lamina propria B cells (A; arrows), TUNEL stain of the same area with arrows pointing out brown apoptotic nuclei (B), and the same area with infiltrating CD11c^+^ macrophages (C) (100×).

First, immunohistologic analyses for apoptosis of B cells in lamina propria areas were performed using TUNEL assays. Apoptotic B cells were frequently seen in the lamina propria often coincident with infiltration of CD68^+^, CD11c^+^ macrophages (Figure 1A–1C).

The most striking changes were noted in the germinal centers of Peyer's patches (Table 3). Initial analysis of terminal ileum tissues in AHI patients not on ART versus those on ART (mean duration of therapy 31 d, Table 1) demonstrated no significant difference in the degree of Peyer's patch follicular lysis, characterized by follicle damage, CD8^+^ T cell and macrophage infiltration, and apoptosis (94.7% in AHI patients not on ART versus 82.6% in AHI patients on ART, Table 3). In terminal ileum tissues from AHI patients not on ART, five of 19 (26.3%) follicles had a germinal center, and this was not significantly different from tissues from AHI patients on ART, in whom ten of 23 (43.4%) had discernable germinal centers. Since similar B cell–inductive microenvironment damage existed in AHI patients both on and not on ART, we combined all AHI patients into a single group for the analyses of terminal ileum using immunohistology and flow cytometry data.

**Table 3:** Germinal center damage in acute HIV-1 infection.

Group	Patients Studied	Total Follicles Studied	Follicular Damage Present	Follicles with Germinal Centers
AHI not on ART	6	19	18/19 (94.7%)	5/19 (26.3%)
AHI on ART^a^	8	23	19/23 (82.6%)	10/23 (43.5%)
AHI total	14	42	37/42 (88.1%)	15/42 (35.7%)
Uninfected	7	32	1/32 (3.1%)	21/32 (65.6%)
			*p*<0.0001^b^	*p* = 0.018^b^

a Duration of ART mean 31 d, range 14–63 d.

b Two-tailed Fisher's exact test for AHI total versus uninfected comparison.

**Figure 2:**
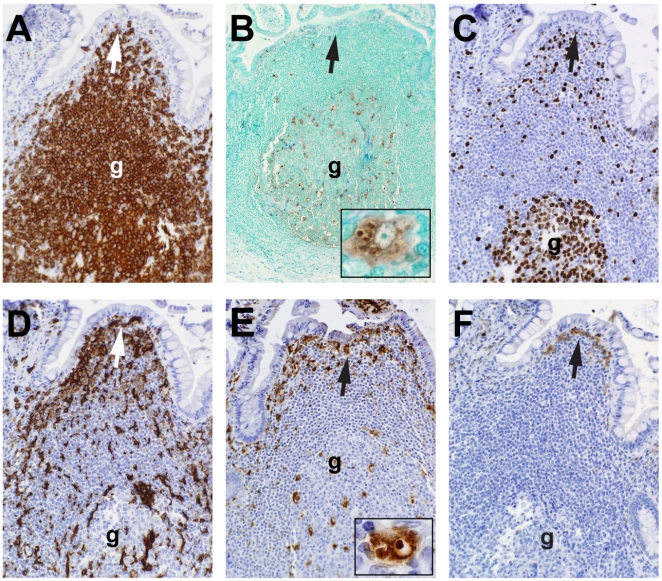
Morphology of uninfected terminal ileum Peyer's patch B cell–inductive microenvironments. Serial sections of the same Peyer's patch follicle in an uninfected terminal ileum (20×). (A) CD20^+^ B cells in a secondary follicle with germinal center (g). (B) The same follicle with TUNEL staining showing that the predominant apoptotic cells are within tingible body macrophages (insert). (C) Ki-67^+^ dividing B cells. (D) CD11c^+^ follicular dendritic cells and macrophages. (E) CD68^+^ macrophages and dendritic cells in the subepithelial zone below follicular associated epithelial cells (arrow, top of photomicrograph) at the dome of the Peyer's patch, and CD68^+^ tingible body macrophages in the germinal center (insert). (F) CD16^+^ NK cells clustered in the areas below follicle associated epithelial cells (arrow).

**Figure 3:**
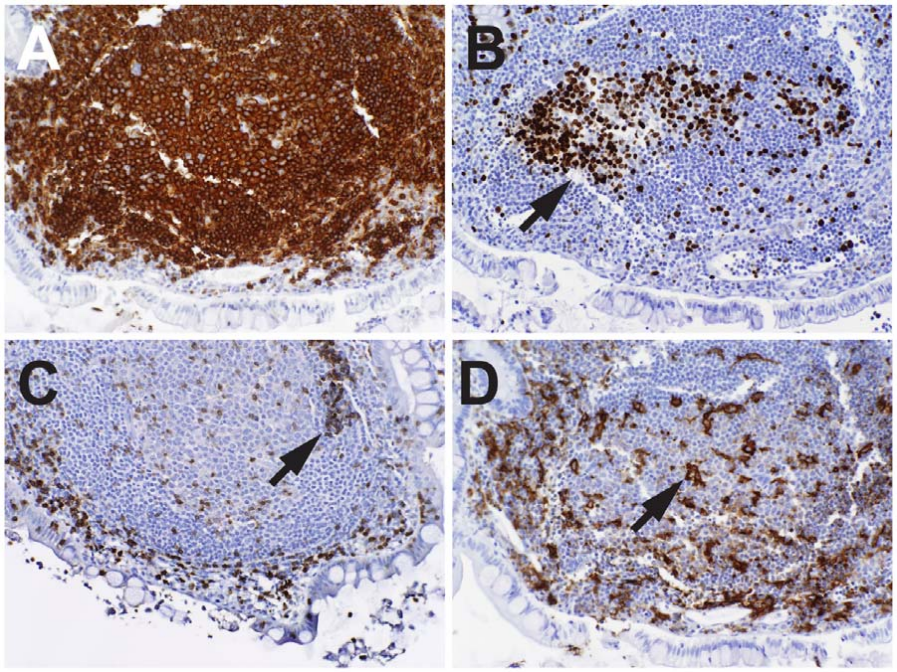
Nearly normal morphology of secondary follicle in AHI terminal ileum. A near-normal terminal ileal secondary follicle in patient 018-4, 74 d after transmission, on ART 29 d (20×). (A) CD20^+^ B cells; (B) Ki-67^+^ dividing germinal center B cells; (C) subepithelial CD3^+^ T cells and a small area of infiltrating CD3^+^ T cells in the marginal zone around the germinal center (arrow); (D) normal-appearing CD11c^+^ macrophages and dendritic cells (arrow). TUNEL stain was negative for apoptotic nuclei (unpublished data).

Overall, in seven terminal ileum tissue samples from uninfected participants, only one of 32 germinal centers showed evidence of follicle cell apoptosis outside of tingible body macrophages, and 21 of 32 (65.6%) B cell follicles contained germinal centers (Figure 2). In contrast, out of 14 AHI patients' tissues analyzed, only 15 of 42 (35.7%) B cell follicles contained germinal centers (*p* = 0.018 for AHI versus uninfected, Fisher's exact test) (Figure 3).

**Figure 4:**
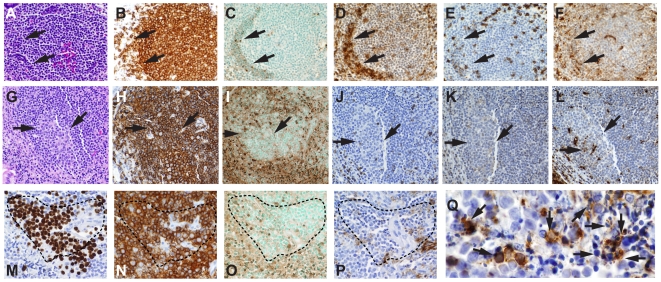
Stages of B cell apoptosis in terminal ileal Peyer's patches soon after HIV-1 transmission. (A–F) Early follicular lysis in a primary follicle of patient 019-2, 47 d after transmission, not on ART; arrows in each photomicrograph show same mantle zone T cell area (A–P, 40×). (A) Hematoxylin and eosin stain. (B) CD20. (C) TUNEL stain with apoptosis in the mantle zone. (D) Rim of CD4^+^ T cells in apoptotic mantle zone. (E) Infiltrating CD8^+^ T cells. (F) Infiltrating CD11c^+^ macrophages. (G–P) Serial sections of a small germinal center remnant in patient 023-3, 66 d after transmission, not on ART (plasma viral load 203 copies/ml, and the only participant in this study who spontaneously controlled plasma viremia). (G) Hematoxylin and eosin stain. (H) CD20^+^ B cells. (I) TUNEL stain of apoptotic B cells surrounding a small germinal center. (J) Follicular CD4^+^ depletion with weakly stained CD4^+^ macrophages. (K) CD8^+^ T cells in follicle areas around the germinal center. (L) CD68^+^ tingible body macrophages in the germinal center (arrows). (M) Ki-67^+^ dividing germinal center cells in serial sections with same area outlined in dotted line; (N) CD20^+^ B cells in same area. (O) TUNEL-stained apoptotic cells surrounding the Ki-67^+^, nonapoptotic germinal center B cells. (P) CD11c^+^ myeloid cells. (Q) Brown CD8^+^ T cells in close apposition to apoptotic and fragmented cells (arrows) in patient 017-9, 56 d after transmission and not on ART (100×).

**Figure 5:**
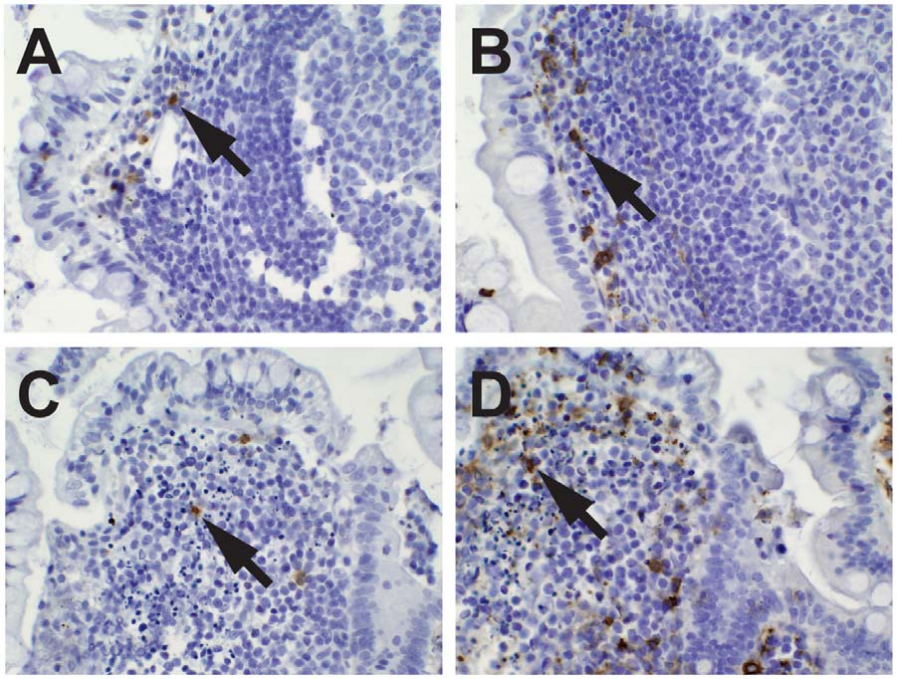
Infiltration of NK cells in apoptotic and nonapoptotic areas of AHI terminal ileum Peyer's patches of patient 018-4. (A and B) The same follicle with germinal center as in Figures 3A–3D; brown CD16^+^ cells (A) and CD56^+^ cells (B). These photomicrographs show normal accumulation of NK cells in subepithelial areas. (C and D) An apoptotic Peyer's patch area in patient 018-4 terminal ileum with brown CD16^+^ cells (C), and CD56^+^ cells (D). In all four panels arrows indicate positive cells (40×).

In the Peyer's patches of uninfected participants, TUNEL^+^ cells were rare, and when present, were phagocytosed by tingible body macrophages within germinal centers (Figure 2B and 2E). In contrast, in Peyer's patches from AHI patients, TUNEL^+^ cells were abundant within the CD20^+^ areas in 13 of 14 patients (Figures 4I, S1B, and S3H). Even in the occasional B cell follicles that contained relatively few TUNEL^+^ B cells, other morphological evidence of programmed cell death (apoptotic bodies, cells with nuclear fragmentation) was clearly apparent either in the follicles or at the follicular margins (Figures 4C, 5C, and 5D). The immunohistology of most HIV-1^+^ terminal ileal follicles was associated with TUNEL^+^ Peyer's patch follicular cells and loss of follicle architecture (Figures 4I, 4O, S1, and S3C–S3H). Twelve of 14 (86%) AHI patients had follicular areas with T cell apoptosis (Figure 4A–4F). In 10 of 14 AHI patients, B-cell follicles contained areas of both B and T cell apoptosis (Figure 4G–4L), and all (14/14) AHI patients had areas of either T cell or B cell apoptosis. When CD205^+^, CD83^+^, and Langerin^+^ dendritic cells were seen, they were either localized in the submucosa below the epithelium (Figure S1A–S1H), or within or around gut epithelium (Figure S2A–S2D).

**Figure 6:**
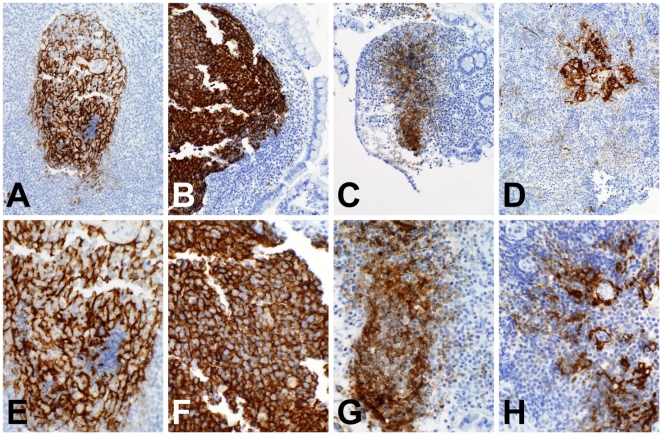
Changes in FDCs) in AHI. (A–D) 20×; (E–H) 40×. (A and E) The fine reticular network of FDCs in an uninfected terminal ileum Peyer's patch (stained with p75 nerve growth factor receptor monoclonal antibody NGFR5). (B and F) Typical hyperplastic FDCs in patient 018-4, 74 d after transmission (stained with NGFR5 monoclonal antibody). (C) (stained with CD21 monoclonal antibody 1F8) and (G) (stained with CD35 monoclonal antibody BerMAC) show condensation and early loss of FDC in AHI terminal ileum 010-1, 52 d after transmission. (D) (stained with NGFR5 monoclonal antibody) and (H) (stained with CD35 monoclonal antibody BerMAC) show progressive FDC destruction in terminal ileum 025-7, 105 d after transmission.

FDC destruction over the course of HIV-1 infection has been well documented, with initial FDC and germinal center hyperplasia followed by FDC destruction and germinal-center involution [32],[33]. To determine how early these changes occurred after HIV-1 infection, staining for FDCs was performed in 12 of 14 terminal ileum samples. We found that 53% of germinal centers had evidence of FDC damage (Figure 6). In contrast, the FDC network in five control tissue samples showed the fine reticular pattern of normal FDCs using monoclonal antibodies to CD21, CD35, and the low-affinity nerve growth factor receptor p75 [23] (Figure 6A and 6E).

Zhang et al. have demonstrated SIV-induced B cell germinal center loss in lymph nodes from rhesus macaques that were poor antibody responders and were rapid progressors to AIDS; there was a concomitant loss of Ki-67^+^ germinal center B cells in samples from these animals [34]. In the AHI participants in our study, whether they were on ART or not, Ki-67^+^ B cells were frequently depleted (Figure 4M–4P), reflecting some degree of germinal center disruption by HIV-1 in most patients studied. When typical germinal centers were present in AHI gut, Ki-67^+^ germinal center B cells were similarly present (Figure 3B).

### Association of Peyer's Patch CD8^+^ T Cells, CD11c^+^ Macrophages, and Natural Killer Cells with Follicular B Cell Apoptosis in Terminal Ileum of AHI Patients

In association with follicular B cell apoptosis, we found infiltration of B cell follicles with CD11c^+^, CD68^+^ macrophages (Figures 4F, 4L, S1C, and S3E), and CD8^+^ T cells (Figures 4E, 4L, 4Q, and S3F), and loss of normal follicle architecture [35],[36]. Overall, there were increased CD8^+^ T cells and CD11c^+^ myeloid cell infiltrations in areas of apoptosis in 10 of 14 patients.

Activated natural killer (NK) cells are expanded in AHI, and their cytolytic activity is correlated with the level of plasma viremia, with a decrease in NK activity after the initiation of ART [37]. While total NK cell numbers are expanded in AHI, there is early depletion of CD56^hi^ CD16^−^ NK cells that secrete cytokines and chemokines [38]; continued viremia causes the remaining cytolytic CD56^dim^ CD16^hi^ NK cells to become anergic. Thus, activated NK cells could be a mediator of B cell death by direct cytolysis or by antibody-dependent cellular cytotoxicity (ADCC), and it was of interest to evaluate B cell follicles for CD16^+^ and CD56^+^ NK cell infiltrations.

In tissues from uninfected participants, Peyer's patches were found to have CD16^+^ NK cells intermixed with CD68^+^ CD11c^+^ macrophages and CD83^+^ dendritic cells at the follicular dome just under the site of follicle-associated epithelial cells (M cells) specialized for antigen transport (Figure 2F) [39], consistent with a recent report by Cella, et al. [40]. B cell areas with massive apoptosis contained only scattered cytolytic CD16^+^, CD57^+^ NK cells [41], but did contain areas of infiltrations of CD56^+^ NK cells (Figures 5A–5D and S1F). Thus, while some degree of B cell apoptosis may be mediated by NK cells, the majority is likely mediated either directly or indirectly by CD8^+^ T cells that were consistently seen infiltrating apoptotic B cell areas (Figure 4Q). CD16^+^ as well as CD57^+^ NK cells were scattered throughout the lamina propria in similar patterns in both uninfected and infected participants (unpublished data).

### Effects of HIV-1 Infection on Numbers of Gastrointestinal Tract Lamina Propria B Cells Using Quantitative Image Analysis

**Figure 7:**
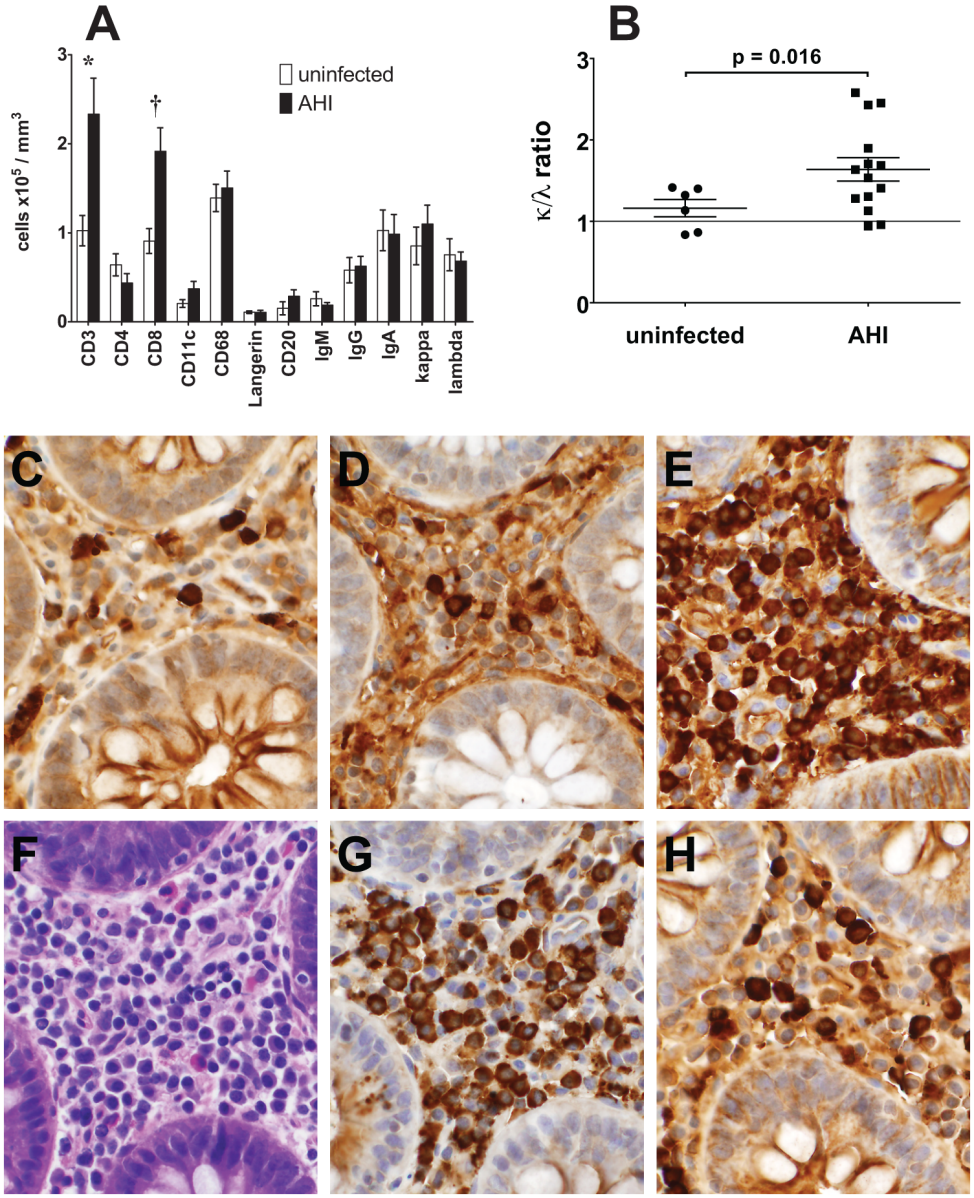
Effect of HIV-1 on Terminal Ileum Immune Cells. (A–B) Bars (A) and horizontal line (B) show mean values; error bars show standard error of the mean. (A) Quantitative image analysis of terminal ileum lamina propria immune cells in six uninfected participants, six AHI patients not on ART, and eight AHI patients on ART. CD3^+^ T cells were elevated in AHI (*p = 0.008, *t*-test) as were CD8+ T cells (^†^ *p* = 0.003, *t*-test). (B) Lamina propria κ/λ ratio skewing (*p* = 0.016, *t*-test). (C–H) (100×) Lamina propria of patient 025-7, 105 d after transmission, on ART 63 d, with predominant IgA, κ plasma cell expansion (κ/λ ratio = 2.53). (C) IgM, (D) IgG, (E) IgA, (F) Hematoxylin and Eosin stain, (G) κ light chains, and (H) λ light chains.

We next evaluated the status of B cells in acute HIV-1 infection in effector (lamina propria) areas in situ in terminal ileum using immunohistochemistry. Figure 7A and Table S2 show the acute HIV-1 induced changes in lamina propria B cell effector sites of terminal ileum. In the lamina propria, there were significant elevations of CD3^+^ T cells (*p* = 0.008) and CD8^+^ T cells (*p* = 0.003, *t*-test) in AHI patients. Lamina propria CD4^+^ T cells were decreased in six of 12 biopsies compared to uninfected terminal ileum biopsies (Table S2).

We studied lamina propria plasma cells for Ig isotype and κ/λ light chain (LC) ratios to better understand plasma cell populations during AHI. AHI patient number 003-7 had the highest number of lamina propria IgG plasma cells and the second highest number of IgA plasma cells, and had a κ/λ LC ratio of 1.7 (Table S2). AHI patient number 025-7 had the highest number of IgA plasma cells and a κ/λ LC ratio of 2.6; Figure 7C–7H show the lamina propria profile of this participant (Table S2). Overall, after a mean of 82 d after transmission the AHI terminal ileum group had an elevated κ/λ LC ratio (1.64±0.14) compared to uninfected terminal ileum (κ/λ = 1.16±0.11; *p* = 0.016, *t*-test) (Figure 7B). Similar κ/λ LC skewing was observed in select Peyer's patch follicles from AHI patients (Figures S3A and S3B).

### Effects of HIV-1 Infection on Percentages of Gastrointestinal Tract B Cells Using Flow Cytometry

**Figure 8:**
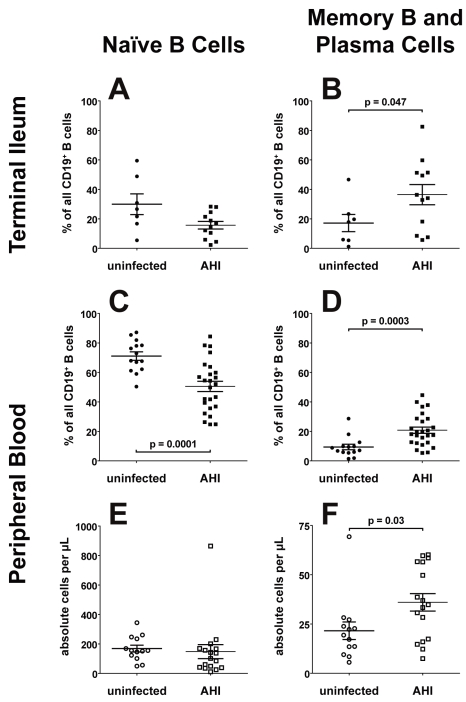
Flow cytometric analysis of B cell populations. For all analyses, B cells were identified as CD3^−^ CD14^−^ CD16^−^ CD235a^−^ and CD19^+^. Horizontal lines show mean values; error bars show standard error of the mean. (A) Terminal ileal naïve B cell percentages. Naïve B cells were defined as B cells that were also surface IgD^+^ and CD27^−^. In cells from terminal ileal biopsies, uninfected participants had a mean±standard error of the mean 29.9%±7.0% of naïve B cells that were decreased to 15.7%±8.9% in AHI patients. (B) Terminal ileal memory and plasma cell percentages. Memory B and plasma cells were defined as B cells that were also surface IgD^−^ and CD27^+^, leaving out IgD^+^ IgM^+^ CD27^+^ B cells. In terminal ileum, uninfected participants had 17.1%±5.8% of memory B and plasma cells that were increased to 36.4%±6.9% in AHI patients (*p* = 0.047, *t*-test). (C) Peripheral blood naïve B cell percentages. Uninfected participants had 71.1%±2.9% of naïve B cells that were decreased to 49.7%±4.2% in AHI patients (*p* = 0.0001, *t*-test). (D) Peripheral blood IgD^−^ CD27^+^ memory and plasma cell percentages. Uninfected individuals had 9.4%±1.9% of memory B and plasma cells that were increased to 20.2%±2.6% in AHI patients (*p* = 0.0003, *t*-test). (E) Absolute numbers of circulating peripheral blood naïve B cells. Calculated as absolute naïve B cells per microliter of blood, uninfected participants had 169±23 cells/µl, while AHI patients had 148±48 cells/µl. (F) Absolute circulating memory B and plasma cells. Calculated as absolute number of cells per microliter of blood, uninfected individuals had 21.6±4.4 cells/µl, and 36.0±4.4 cells/µl in AHI participants (*p* = 0.03, *t*-test). All data in this figure were also analyzed using a two-tailed exact Wilcoxon test that resulted in similar *p*-values.

We next used flow cytometry to determine the percentages of total terminal ileum B cells in tissues from AHI patients compared to uninfected control tissues. In mononuclear cells, we found an increase in the percentage of B cells in AHI terminal ileum (26.2%) compared to control uninfected terminal ileum (12.5%) (*p* = 0.006, *t*-test). There were no differences between AHI patients who were on or not on ART (25.3% versus 27.9%, respectively; mean duration of therapy 31 d, range 14–63 d; Table 1). There was no significant difference in naïve B cells between AHI and uninfected participants (Figure 8A) but the percentages of gut memory B plus plasmablasts/plasma cells (CD19^+^ IgD^−^ CD27^+^) was elevated in AHI versus uninfected participants (Figure 8B) (*p* = 0.047, *t*-test). There were no differences in naïve, memory, or plasma cell subsets between AHI patients who were on or not on ART (unpublished data).

### Effect of HIV-1 on Peripheral Blood B Cell Subsets

We next used the same flow cytometric method to study B cell subsets in peripheral blood in AHI versus uninfected participants. The percentages and absolute numbers of total blood B cells in AHI patients not on ART (*n* = 12) versus AHI patients on ART (*n* = 9) were not different (4.0%, 195 B cells/µl versus 5.5%, 326 B cells/µl, respectively). Similarly, no differences were seen in total blood B cell absolute numbers/µl in AHI versus uninfected participants (3.7%, 227 B cells/µl in uninfected participants) (Table S3).

In contrast to reports of elevated numbers of blood naïve and transitional B cells in the blood of patients with chronic HIV-1 infection [11]–[13], naïve B cells in the blood of patients with acute and early HIV-1 infection were markedly decreased as a percentage of the total B cell compartment (*p* = 0.0001, *t*-test) (Figure 8C). Calculated as absolute number of circulating naïve B cells/µl of blood, we found no significant difference in naïve B cell number between uninfected and AHI participants (Figure 8E). The combined percentages and absolute numbers of memory B cells and plasmablasts/plasma cells were elevated in the blood of AHI patients (*p* = 0.0003 and *p* = 0.03, respectively, *t*-test) (Figures 8D and 8F). Elevations of terminally differentiated B cells in AHI were observed at the earliest time points studied, 17–36 d after transmission, where the percentage of memory B and plasma cells was 23.7%±3.3% (range 12.4%–37.9%) compared to 9.4%±1.9% in the uninfected control participants. Analyzed separately, the percentages of both memory B cells and plasmablasts/plasma cells in the blood of AHI patients were elevated compared to uninfected control participants (*p* = 0.005 and *p* = 0.005, respectively, *t*-test) (Figure S4B and S4E). Absolute number of circulating memory B cells were elevated in AHI patients (*p* = 0.01, *t*-test), whereas circulating plasmablasts/plasma cells were similar to uninfected controls (Figure S4C and S4F).

Analysis was also performed for all of the B cell subsets described above by separating participants into high plasma VL and low VL groups. No significant differences were found for B cell subsets between the two groups (*t*-test).

Recently, a population of CD19^+^ IgM^+^ CD27^+^ B cells has been described as IgM^+^ memory B cells and/or circulating human marginal zone B cells [42]–[44]. CD19^+^ IgD^−^ IgM^+^ CD27^+^ cells were included in the above memory B cell analysis, but we did not analyze the CD19^+^ IgD^+^ IgM^+^ CD27^+^ B cell subset. A separate analysis of this population showed that the percentage of CD19^+^ IgD^+^ IgM^+^ CD27^+^ B cells in blood was similar in both uninfected and AHI cohorts (7.0%±1.5% in uninfected, 6.2%±1.5% in AHI, not significantly different, *t*-test). In terminal ileum, CD19^+^ IgD^+^ IgM^+^ CD27^+^ B cell percentages also were not different between infected and uninfected participants (1.9%±0.7% in uninfected, 1.4%±0.3% in infected, not significantly different, *t*-test). Finally, including CD19^+^ IgD^+^ IgM^+^ CD27^+^ B cell populations in our analyses did not alter our conclusion that acute HIV-1 infection results in increased percentages of memory cells in blood or gut (unpublished data).

### EBV Transformation of Terminal Ileum Memory B Cells for Analysis of Specificity of Antibodies Produced

**Figure 9:**
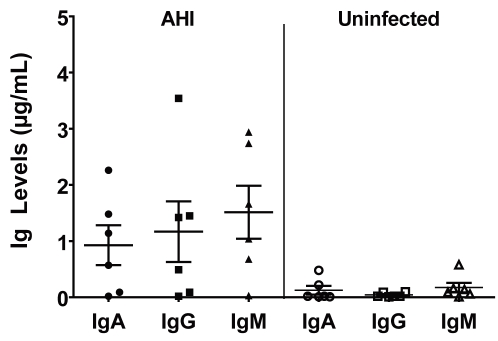
Total immunoglobulin (Ig) levels and specificities in the EBV-transformed B cell cultures. Horizontal lines show mean values; error bars show standard error of the mean. Graph shows the levels of IgA, IgG, and IgM in the cell culture supernatants derived from terminal ileal samples of six acute/early HIV-infected and six uninfected participants determined by ELISA 14 d after EBV transformation. Each data point represents the geometric mean of IgA, IgG, and IgM levels in all wells plated from each terminal ileum sample.

EBV preferentially transforms recently primed B cells [45]. The amount of IgM, IgG, or IgA produced in 96-well cultures of EBV-transformed B cells was determined in six AHI terminal ileum samples versus terminal ileum from six uninfected participants. If HIV-1 infection promoted class-switching there should be an increased number of primary EBV-transformed cultures producing supernatant IgG and IgA after EBV transformation compared to terminal ileum B cell cultures from uninfected individuals. In addition, if there are more primed IgM^+^ B cells present in terminal ileum, there should be more B cell cultures with elevated IgM. Figure 9 shows that this was the case with more IgM-, IgG-, and IgA-producing B cell cultures in AHI terminal ileum 2 wk after EBV stimulation versus uninfected EBV-transformed B cell cultures.

Next, we determined the relative frequency of the specificities of antibodies in each EBV-transformed well of B cells to determine the relative percentages of B cells responding to HIV-1, either as HIV-1–specific or as polyclonally induced antibodies (Table 4). This method of analysis does not reflect the absolute percentage of memory B cells making various antibody specificities, because the gut mononuclear cells were cultured at 10,000 cells per well as opposed to single cell per well cultures. Rather this analysis provides the relative frequency of antibody specificities in the entire terminal ileum memory B cell population. In uninfected participants, 8/420 (1.9%) of EBV-transformed B cell cultures were positive for gp41 antibodies, and 1.9% were positive for gp120 antibodies (IgM only); none were positive for antibodies to HIV-1 Tat, while 1.4% and 0.2% were positive for anti-cardiolipin antibodies or rheumatoid factor, respectively (Table 4). Two percent of cultures from uninfected participants were also positive for reactivity with the 2007 inactivated influenza vaccine Fluzone (Sanofi Pasteur). In contrast, in AHI terminal ileum, 18.6% of EBV B cell cultures were positive for antibodies to HIV-1 gp41, 5.7% for antibodies to HIV-1 gp120, and 9.3% for antibodies to HIV-1 Tat. In addition, 6.2% of AHI B cell cultures were reactive with cardiolipin, 3.0% had rheumatoid factor activity and 16.2% were reactive with the 2007 inactivated influenza vaccine (Table 4). Taken together this pattern of relative specificities of EBV-transformed memory B cells indicated the presence of a large component of polyclonal B cell activation in addition to HIV-1–induced specific antibody responses.

**Table 4:** Percentage of EB virus-stimulated cultures producing antibodies reactive with HIV and non-HIV antigens.

Group	Antibody Specificity					
	gp41	p120	Tat	Cardiolipin	Rheumatoid Factor	Fluzone
Acute/Early HIV Infection (*n* = 8)	18.6% (108/580)	5.7% (17/296)	9.3% (37/396)	6.2% (22/353)	3.0% (9/296)	16.2% (22/136)
Uninfected (*n* = 4)	1.9% (8/420)	1.9% (8/420)	0% (0/420)	1.4% (6/420)	0.2% (1/420)	2.1% (9/420)
Fisher's exact test	*p*<0.0001	*p* = 0.007	*p*<0.0001	*p* = 0.0004	*p* = 0.0021	*p*<0.0001

Table shows the percentage of wells of terminal ileum cells seeded at 10^4^ cells/well and assayed 21–28 d after EBV transformation. Percentages represent positive wells out of number of cultures indicated that were assayed for antibodies reactive with gp41, gp120, Tat, CL, and killed influenza in the 2007 influenza vaccine Fluzone, as well as RF. The comparison group was a total of 420 cultures from four uninfected participants.

### Relationship of Plasma and GALT HIV-1 Viral Load to Peyer's Patch Germinal Center Morphology

We identified Peyer's patch follicles containing germinal centers at various stages of damage in eight AHI patients (Figure 3A–3D; Table 1). Comparison of GALT germinal center depletion and plasma VL demonstrated that those with germinal center depletion had significantly higher viral loads (mean 34,065 copies/ml) than those with germinal centers (mean 1,711 copies/ml) (*p* = 0.021, exact Wilcoxon test). Six of eight patients with germinal centers had initiated ART, while only two of six of the patients with no germinal centers were on ART. These data are in contrast to the work of Muro-Cacho et al. who showed that the intensity of lymph node apoptosis in advanced HIV-1 infection did not correlate with plasma VL [46]. It is of interest that, of the 14 participants with gut biopsies, the only patient (023-3) that controlled plasma viremia in the absence of ART (203 copies/ml at the time of sampling 66 d after transmission) also had some degree of germinal center preservation (Figure 4G–4P). GALT-associated HIV-1 RNA did not correlate with germinal center depletion (Table 5) [47].

**Table 5:** Comparison of AHI patients with terminal ileum germinal centers versus those without terminal ileum germinal centers.

Parameter	With Terminal Ileum Germinal Centers^a^	Without Terminal Ileum Germinal Centers
Number of patients	8	6
Plasma viral load (RNA copies/ml, mean^b^ [range])	1,711 (<50–387,344)^c^	34,065 (6,570–123,928)^c^
GALT viral load (RNA copies/ml, mean^b^ [range])	324 (<50–10,639)	449 (38–44,816)
CD4 cells in PBMC (cells/µl)	735	609
CD4 cells in tissue (cells/mm^3^)	49,708	31,648
CD8 cells in PBMC (cells/µl)	1,174	1,046
CD8 cells in tissue (cells/mm^3^)	184,162	201,564
Estimated time from transmission, d	80	81
Patients on ART	6/8	2/6
Days on ART prior to biopsy	36 d	16 d
κ/λ LC ratio	1.63	1.65

a Patients 019-2, 023-3, 008-6, 010-1, 016-8, 018-4, 025-7, 016-2 from Table 1.

b Plasma viral load and GALT viral load are calculated as geometric means.

c *p* = 0.021, exact Wilcoxon test, comparing patients with terminal ileum germinal centers versus those without terminal ileum germinal centers.

### Effect of Presence of Peyer's Patch Germinal Centers and Antiretroviral Treatment During Acute HIV-1 Infection on Plasma Rheumatoid Factor Levels and HIV-1 Protein Antibody Levels

Comparison of HIV-1 envelope and gag p55 antibodies in patients with germinal centers to those with no germinal centers showed no differences except for higher anti-gp41 IgG antibodies in those without germinal centers (*p* = 0.005, *t*-test) (Table 6). Similarly, there was no difference in antibody levels when the participants providing terminal ileum biopsies (Table 1) were grouped as high- (>20,000 copies/ml) versus low plasma VL (<20,000 copies/ml) (unpublished data). We next determined the effect of early institution of ART on levels of plasma IgM, IgG and IgA antibodies against HIV-1 gp41, p55, gp120 and gp140, as well as plasma rheumatoid factor levels, in patients from whom terminal ileal biopsies were obtained (Table 7). The time from transmission in the treated versus the non-treated group was similar (82 d versus 79 d) (Table 1). As expected, the anti-gp41 IgA, anti-gp140 IgA, and anti-gp140 IgG levels were significantly lower in the AHI on ART group compared to the AHI on no ART group. Thus, even one month of ART significantly lowered plasma levels of antibody to HIV-1 Env following HIV transmission.

**Table 6:** Antibody responses to HIV-1 proteins: Antibody to HIV-1 proteins in AHI with germinal centers versus no germinal centers.

Group	Antibody Specificity (µg/ml Equivalent)				
	gp41	p55	gp120	gp140	Rheumatoid Factor
AHI with terminal ileum germinal centers (*n* = 8)					
IgM	3.8	3.6	0.6	0.1	0.3
IgG	22.0^a^	30.2	3.7	1.5	N/A
IgA	2.9	2.1	1.2	1.0	N/A
AHI without terminal ileum germinal centers (*n* = 6)					
IgM	1.1	6.3	1.4	0.5	0.5
IgG	39.6*	40.1	6.7	7.1	N/A
IgA	4.1	3.4	1.2	1.2	N/A

a gp41 antibodies are higher for patients without germinal centers (*t* = 3.62, *p* = 0.005) by unequal variance *t*-test.

N/A, not applicable.

**Table 7:** Effect of antiretroviral treatment in AHI on HIV-1 antibody responses.

Group	Antibody Specificity (µg/ml Equivalent)				
	gp41	p55	gp120	gp140	Rheumatoid Factor
AHI not on ART (*n* = 6)					
IgM	1.7	6.4	0.5	0.8	0.5
IgG	36.0	29.7	5.7	7.1^a^	N/A
IgA	5.4^b^	2.3	1.2	1.7^c^	N/A
AHI on ART (*n* = 8)					
IgM	3.4	3.5	1.1	1.0	0.4
IgG	24.8	38.1	4.4	1.5^a^	N/A
IgA	1.9^b^	2.9	1.2	0.6^c^	N/A

Responses lower in AHI on ART group versus AHI not on ART group by unequal variance *t*-test (^a^IgG gp140, *p* = 0.047; ^b^IgA gp41, *p* = 0.020; ^c^IgA gp140, *p* = 0.020).

N/A, not applicable.

## Discussion

By as early as 17 d after transmission, HIV-1 infection induced B cell class switching manifested by acute reductions in numbers of naïve B cells and striking elevations of memory B cells and plasmablasts/plasma cells in blood and terminal ileum. By 47 d after transmission, HIV-1 infection was associated with damage to GALT germinal centers, including follicular lysis due to B and T cell apoptosis as well as destruction of the FDC networks in terminal ileum Peyer's patches.

### HIV-1-Induced Polyclonal B Cell Activation

In this study we document, to our knowledge for the first time, polyclonal B cell activation in terminal ileum in early HIV-1 infection. HIV-1–induced polyclonal B cell activation has been well documented in chronic HIV-1 infection, and several studies have used assays to characterize unstimulated (or spontaneous) secretion of anti-HIV-1 antibody by circulating B cells during the first six months after HIV-1 infection [7]–[12],[14],[15],[48]. Morris et al. reported increased frequencies of B cells that (spontaneously) secreted Ig in blood of patients chronically infected with HIV-1, and also demonstrated elevated levels of B cells that (spontaneously) produced specific antibody to gp120 [14]. The same group also assayed blood from three patients recently infected with HIV-1 and found elevated numbers of antibody-secreting cells specific for Env gp120 [14]. The mechanism of HIV-1 gp120-induced polyclonal B cell activation in chronic HIV-1 infection may be mediated by cytokines such as IL-15 and TNF-α [49],[50], by translocation of bacteria and lipopolysaccharide across gut barriers [51], by gp120 binding as a superantigen to VH3 Ig^+^ B cells [52], and/or gp120 binding to mannose C-type lectin receptors on a subset of B cells [53]. Whether these mechanisms of B cell activation are operative in early HIV-1 infection remains to be determined.

### Follicular Damage and Germinal Center Loss in HIV-1 Infection

The germinal center loss seen in terminal ileum may be more pronounced early on after infection than has been previously reported in lymph nodes and spleen, where germinal center hyperplasia is common in AHI [54],[55]. Germinal center hyperplasia was seen in our study in AHI terminal ileum, but significant germinal center destruction was also present that correlated with plasma viral load. One difference may relate to the massive and early CD4^+^ T cell loss that is so prominent in gut tissues [1]–[5]. The early loss of gut B cell germinal centers may impair effective humoral responses to HIV-1. CD4^+^ T cell help is required for induction of long-lived plasma cells and long-term production of high-affinity antiviral antibodies [56]. Thus, given early damage and/or loss of GALT follicles with germinal centers, one expectation would be that the apparent half-life of induced antibody to HIV-1 would be short (weeks to months) and not reflect a level maintained by long-lived plasma cells (∼10 y or longer) [57]. This hypothesis is indeed consistent with the reported apparent half-life of anti-p24 and anti-gp120 antibodies in chronically infected patients of 9–15 wk and 7–21 wk, respectively [14]. The destruction of germinal centers due to inflammation and the lack of production of neutralizing antibodies have also been reported for another model of chronic viral infection, lymphocytic choriomeningitis virus, where chronic CD27-mediated stimulation of T cells by CD70 on B cells resulted in cytokine-mediated germinal center destruction [58]. It will be of interest in future studies to determine if this mechanism is responsible for the destruction seen in acute HIV-1 infection in humans.

Pauza and colleagues demonstrated loss of peripheral B cells in monkeys with acute SIV infection that rapidly progressed to AIDS in the absence of seroconversion [59],[60], and Zhang et al. have recently demonstrated loss of germinal centers in three additional SIV^+^ rhesus macaques that progressed rapidly to AIDS [34]. A previous study of B cells in patients with primary HIV-1 infection showed decreases in total peripheral blood B cell levels by 2 mo after transmission [15]. That study went on to show elevated levels of apoptotic naïve B cells in early HIV-1 infection, with levels of naïve and memory B cells similar to infected controls. Our study demonstrated no significant difference in blood total B cell numbers in acute HIV-1 infection (Table S3) and striking elevations in memory and plasma cells not seen in prior work. These findings are likely due to the short time since HIV-1 infection of participants in the present study.

Follicular lysis of mantle zones of lymph node follicles in chronic HIV-1 infection, first described in 1985, consists of destruction of FDCs in germinal centers, loss of germinal center morphology, and germinal center infiltration with CD8^+^ T cells and macrophages [35],[36]. Early studies described an increase in lymph node CD1^+^ Langerhans dendritic cells in the paracortex of chronic HIV-1 lymph nodes [35],[36]. In contrast, in AHI in Peyer's patches and lamina propria of terminal ileum, we saw no increase in CD83^+^ or Langerin^+^ dendritic cells, but saw striking infiltrations of apoptotic follicular areas with infiltration of CD8^+^ T cells and CD11c^+^ CD68^+^ macrophages.

### Mechanisms of Damage to Terminal Ileum Inductive Microenvironments

The mechanisms of terminal ileum Peyer's patch B cell apoptosis in AHI are yet to be fully determined. However, CD8^+^ T cell infiltration has been well documented in HIV-1 lymph nodes [54] and thymus [61], and lymph node CD8^+^ T cells are armed for effector formation [62]. The close association of CD8^+^ T cells with apoptotic cells in B cell areas (Figure 4Q), accompanied by increased numbers of CD8^+^ T cells in blood and terminal ileal biopsies from patients who exhibit extensive germinal center loss (Table 4) suggests CD8-mediated B cell killing. The infiltration of CD11c^+^ CD68^+^ macrophages into apoptotic B cell areas may represent the clearing of apoptotic cells and cell debris by phagocytosis, or, alternatively, activation of macrophage-dependent cytotoxicity such as ADCC. From the patterns of CD16^+^ CD57^+^ NK effector cells seen infiltrating lymphoid follicles, NK cells as predominant effectors of killing of follicle B cells seems unlikely. B cell apoptosis could also result from changes in positioning cues, e.g., inflammatory or homeostatic cytokines, caused by infection and/or the apoptotic process per se. We have previously shown early elevations in plasma tumor necrosis factor apoptosis-inducing ligand (TRAIL) in AHI before peak viral load, and B cell death within germinal centers could reflect TRAIL-mediated bystander B cell killing [63]. Fas ligand and tumor necrosis receptor type II (TNFRII) plasma levels are also elevated soon after TRAIL in AHI [63] as are plasma TNF-α levels [64]—all of which could contribute to B cell death. That B cell apoptosis is localized to B cell areas surrounding germinal centers and where HIV-1 is localized [47],[65] also suggests that HIV-1 may trigger B cells to activation-induced cell death after antigen presentation by follicular dendritic cells.

Persistent activation of B cells by HIV-1 likely results in large populations of B cells that soon require T cell help for their continued survival [66]–[70], help that is unavailable because antigen-specific T helper cells are not present or have been lost to infection and cell lysis. In the absence of cognate T cell help, B cells generally die within a few days of their activation [66]–[70]. This physiologic requirement for the interaction of antigen-specific T- and B lymphocytes ensures that under physiologic conditions long-lived B cells, memory B cells, and plasma cells resident in bone marrow are regulated by the stringent mechanisms that control T cell tolerance [71],[72]. This model can explain the rapid follicular lysis and losses of naïve B cells associated with acute HIV-1 infection.

Given the ability of HIV-1 to induce polyclonal B cell activation, our observation of skewed κ/λ LC ratios in GALT tissue from AHI patients is intriguing. We have recently sorted and sequenced Ig genes from a total of over 700 plasmablasts/plasma cells from blood of three AHI patients. In these individuals, the κ/λ LC ratios were similarly elevated as assayed by direct Ig sequencing of sorted plasmablasts/plasma cells (unpublished data). Interestingly, similar κ/λ LC ratio skewing has been reported in responses to gp120 in SIV-infected rhesus macaques [73], and in the response to HIV-1 gp120, p24, and reverse transcriptase in HIV-1 infection in humans [74].

There are several limitations to our study that warrant further work. First, of necessity we obtained the terminal ileum biopsies from limited areas and they may not be fully representative of all gut areas. Additional work may reveal a more detailed picture of early HIV-1–induced immune system damage. EBV transformation of memory B cells can skew the B cell repertoire, such that our comparison of uninfected and AHI participants is only qualitative. Finally, heterogeneity of patient responses to both HIV-1 infection and ART can confound data analysis. These issues not withstanding, the damage to B cell–inductive microenvironments and early polyclonal B cell differentiation in HIV-1 infection are apparent from the present analyses.

### Conclusion

Early HIV-1 infection induces germinal center damage and loss as well as polyclonal activation of gut B cells. These data suggest that for an HIV-1 vaccine to be effective, it will need to induce significant levels of broadly neutralizing antibodies that are present before HIV-1 transmission. If transmission is not prevented by preexisting antibody, then an effective AIDS vaccine will also need to prime for rapid (hours to days) innate responses of anti-HIV-1 factors (such as defensins [75], alpha-1-antitrypsin [76], CCR5 binding chemokines [77], or anti-HIV-1 NK cell responses [78],[79]) that might protect against HIV-1–induced follicular germinal center B cell and CD4^+^ T cell loss until robust CD8^+^ T cell and secondary B cell responses are induced.

## Supporting Information

Figure S1 Subepithelial remnant of a B cell germinal center in patient 023-3, 66 d after transmission, not on ART showing a rare cluster of CD83^+^ and CD205^+^ dendritic cells. (A) CD20^+^ B cells; (B) TUNEL^+^ apoptotic cells; (C) CD11c^+^ infiltrating macrophages; (D) few of the CD11c^+^ cells are CD83^+^ dendritic cells (arrow); (E) scattered CD205^+^ dendritic cells (arrows); (F) scattered CD16^+^ NK cells (arrow); (G) scattered CD57^+^ cells (arrow); (H) rare Ki-67^+^ cells (arrow) (40×).
(4.7 MB TIF)

Figure S2 Langerin^+^ dendritic cells, when present, were associated with epithelial cells. (A–D) Patient 023-3 66 d after transmission not on ART. Arrows show Langerin^+^ dendritic cells in and around epithelial cells of terminal ileum villi (100×).
(4.6 MB TIF)

Figure S3 Ig light chain staining of Peyer's patch in patient 008-6 and extensive B cell destruction in patient 020-4. (A and B) Kappa light chain predominance in a Peyer's patch follicle in patient 008-6, 67 d after transmission, on ART 17 d. Figure S4A shows a follicle area with brown kappa positive cells in center of tissue, with the same area in the sequential section (B) with fewer lambda light chain B cells present (10×). (D–H) Extensive B cell destruction in serial Peyer's patch areas of patient 020-4, 113 d after transmission, not on ART. (C) CD20^+^ B cells; (D) scattered Ki-67^+^ cells (arrows); (E) mass of CD11c^+^ macrophages in the same area; (F) CD8^+^ T cells infiltrating apoptotic area; (G) scattered CD16^+^ NK cells; (H) TUNEL stain showing cells with apoptotic nuclei in brown (40×).
(7.3 MB TIF)

Figure S4 Flow cytometric analysis of B cell populations. As in Figure 1, for all analyses, B cells were identified as CD3^−^ CD14^−^ CD16^−^ CD235a^−^ and CD19^+^. (A) Terminal ileal memory B cell percentages. Memory B cells were defined as B cells that were also surface IgD^−^, CD38^−/+^, and CD27^+^. In cells from terminal ileal biopsies uninfected participants had 12.9%±5.4% of memory B cells that were increased to 26.8%±5.4% in AHI patients. (B) Peripheral blood memory B cell percentages. Uninfected participants had 7.3%±1.4% of memory B cells that were increased to 13.1%±1.6% in AHI patients (*p* = 0.005). (C) Absolute numbers of circulating memory B cells. Calculated as absolute memory B cells per microliter of blood, uninfected participants had 15.4±2.0 cells/µl that were increased to 27.0±3.7 cells/µl in AHI patients (*p* = 0.01). (D) Terminal ileal plasma cell percentages. Plasma cells were defined as B cells that were also surface IgD^−^, CD38^hi^, and CD27^+^. In terminal ileum, uninfected participants had 4.2%±1.5% of plasma cells that were increased to 9.6%±3.2% in AHI patients. (E) Peripheral blood plasma cell percentages. Uninfected participants had 2.1%±0.8% of circulating plasma cells that were increased to 7.1%±1.9% in AHI patients (*p* = 0.005). (F) Absolute circulating plasma cells. Uninfected participants had circulating plasma cells at 6.2±3.1 cells/µl while AHI patients had 9.0±1.8 cells/µl. Absolute cell number data (Figure S4C and S4F) were also analyzed using a two-tailed exact Wilcoxon test resulting in similar *p*-values.
(0.52 MB EPS)

Table S1 Antibodies and their source used in immunohistologic analysis.
(0.06 MB DOC)

Table S2 Terminal ileal biopsy histologic data.
(0.1 MB DOC)

Table S3 Total B cell populations in blood in acute HIV-1 infection.
(0.04 MB DOC)

Text S1 Additional methods.
(0.04 MB DOC)

## Abbreviations

ADCC: antibody-dependent cellular cytotoxicity
AHI: acute HIV-1 infection
ART: antiretroviral treatment
FDC: follicular dendritic cell
EBV: Epstein-Barr virus
GALT: gut-associated lymphoid tissue
LC: light chain
NK: natural killer
VL: viral load
