# Supplementary figures and images for: Polyclonal B Cell Differentiation and Loss of Gastrointestinal Tract Germinal Centers in the Earliest Stages of HIV-1 Infection
Source: PLoS Med. 2009 Jul 7;6(7):e1000107. doi: 10.1371/journal.pmed.1000107 (PMC2702159; doi:10.1371/journal.pmed.1000107)

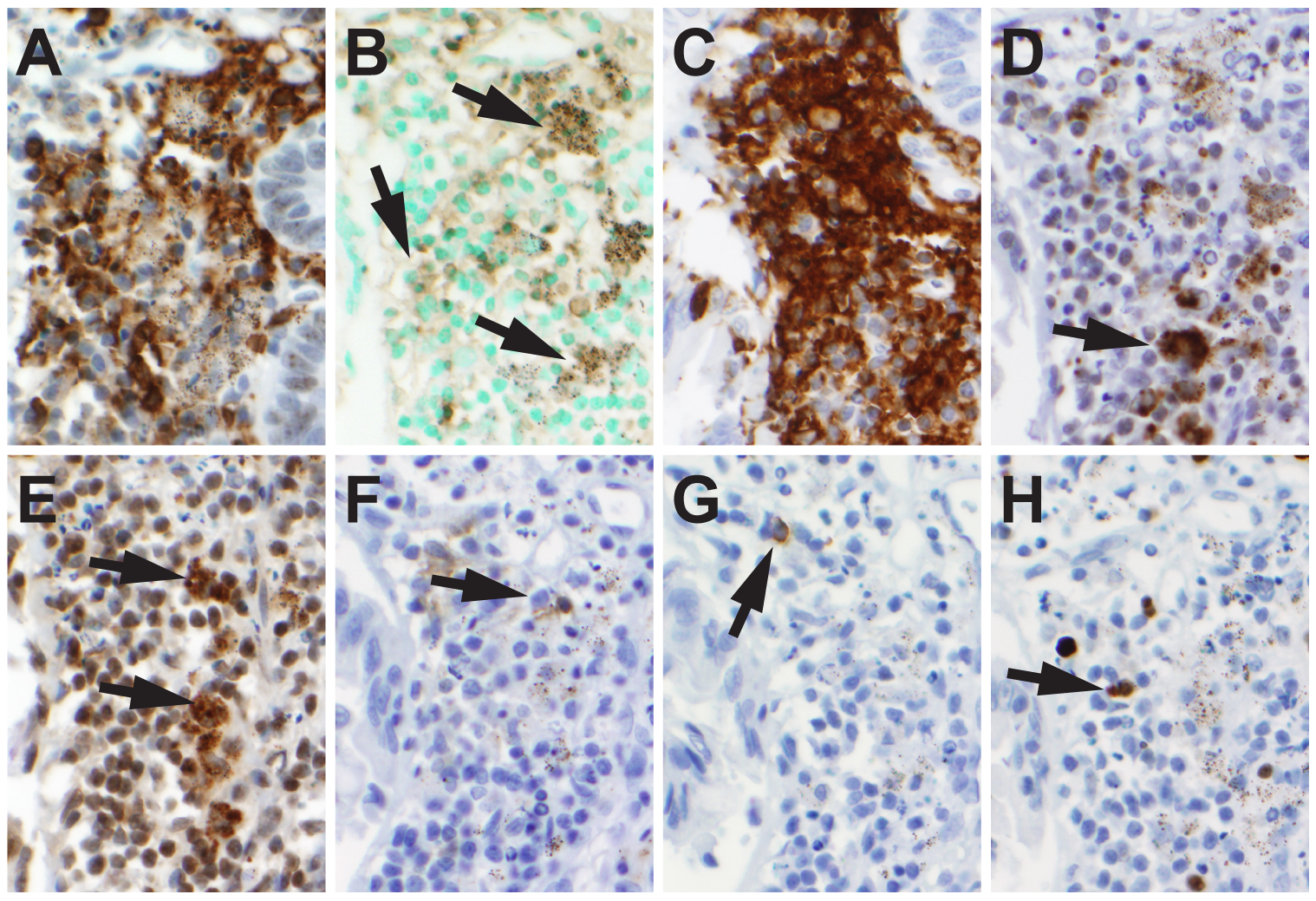

Supplement: Figure S1 — Subepithelial remnant of a B cell germinal center in patient 023-3, 66 d after transmission, not on ART showing a rare cluster of CD83+ and CD205+ dendritic cells. (A) CD20+ B cells; (B) TUNEL+ apoptotic cells; (C) CD11c+ infiltrating macrophages; (D) few of the CD11c+ cells are CD83+ dendritic cells (arrow); (E) scattered CD205+ dendritic cells (arrows); (F) scattered CD16+ NK cells (arrow); (G) scattered CD57+ cells (arrow); (H) rare Ki-67+ cells (arrow) (40×). (4.7 MB TIF) [file pmed.1000107.s001.tif]

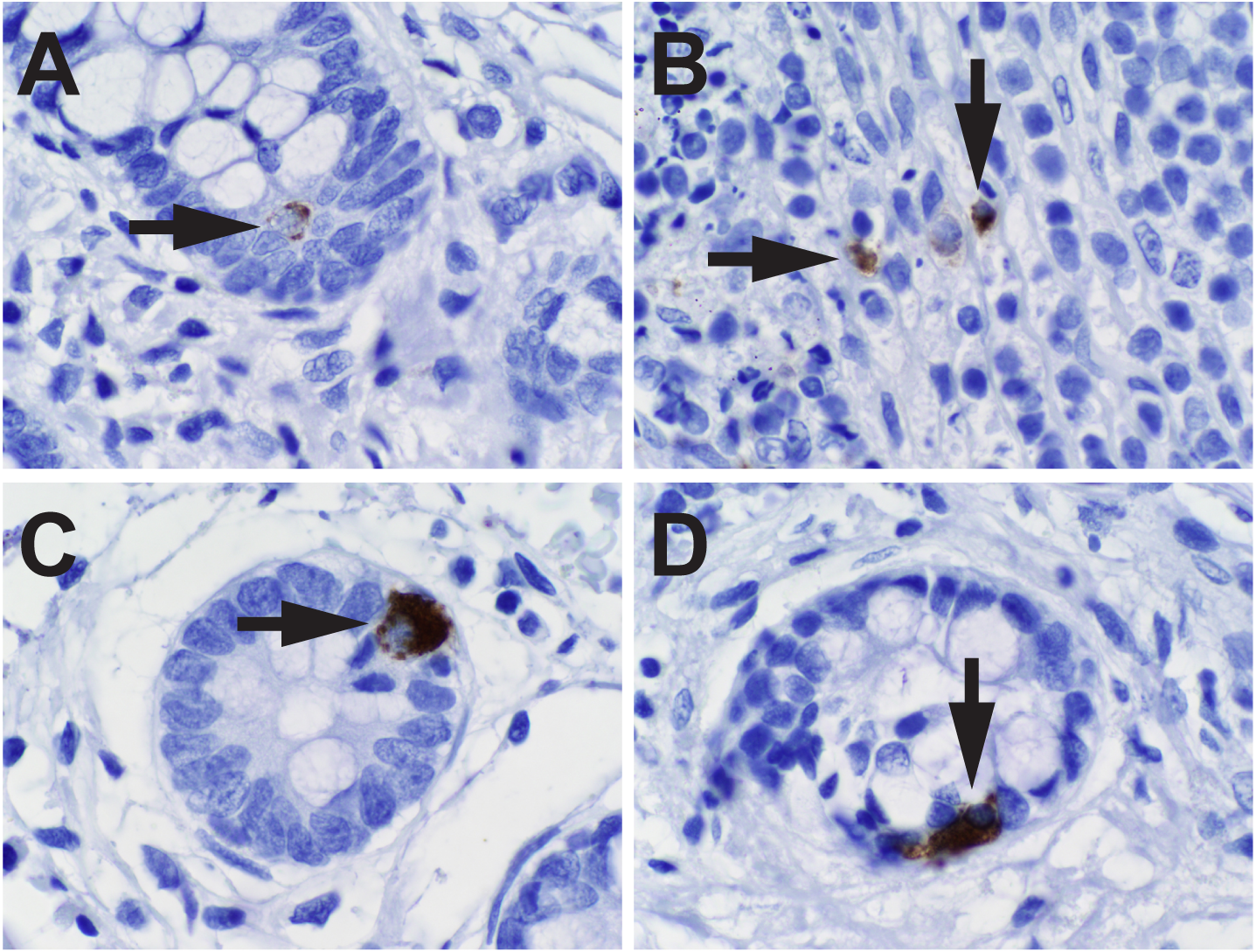

Supplement: Figure S2 — Langerin+ dendritic cells, when present, were associated with epithelial cells. (A–D) Patient 023-3 66 d after transmission not on ART. Arrows show Langerin+ dendritic cells in and around epithelial cells of terminal ileum villi (100×). (4.6 MB TIF) [file pmed.1000107.s002.tif]

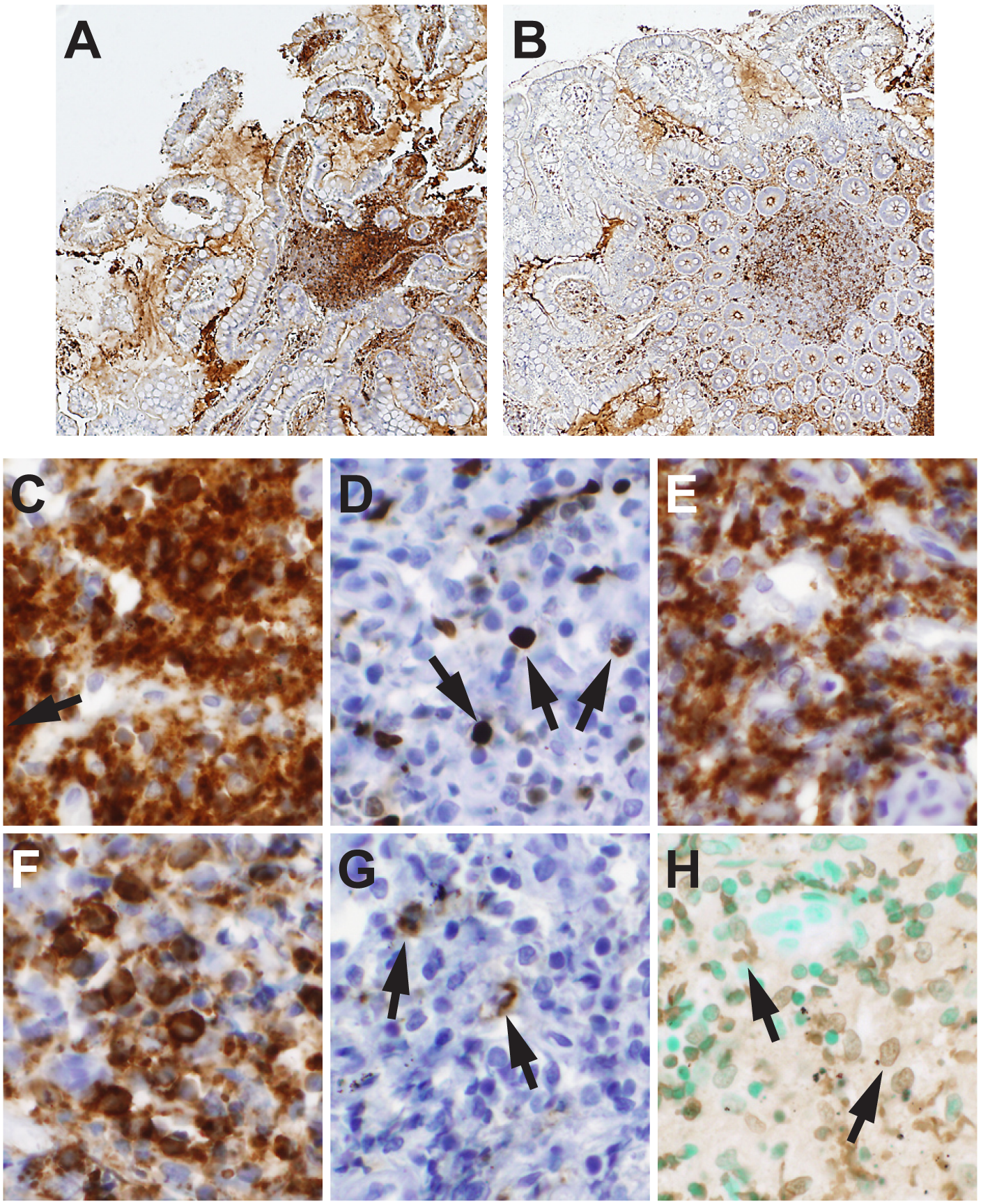

Supplement: Figure S3 — Ig light chain staining of Peyer's patch in patient 008-6 and extensive B cell destruction in patient 020-4. (A and B) Kappa light chain predominance in a Peyer's patch follicle in patient 008-6, 67 d after transmission, on ART 17 d. Figure S4A shows a follicle area with brown kappa positive cells in center of tissue, with the same area in the sequential section (B) with fewer lambda light chain B cells present (10×). (D–H) Extensive B cell destruction in serial Peyer's patch areas of patient 020-4, 113 d after transmission, not on ART. (C) CD20+ B cells; (D) scattered Ki-67+ cells (arrows); (E) mass of CD11c+ macrophages in the same area; (F) CD8+ T cells infiltrating apoptotic area; (G) scattered CD16+ NK cells; (H) TUNEL stain showing cells with apoptotic nuclei in brown (40×). (7.3 MB TIF) [file pmed.1000107.s003.tif]
