# Supplementary material for: Polyclonal B Cell Differentiation and Loss of Gastrointestinal Tract Germinal Centers in the Earliest Stages of HIV-1 Infection
Source: PLoS Med. 2009 Jul 7;6(7):e1000107. doi: 10.1371/journal.pmed.1000107 (PMC2702159; doi:10.1371/journal.pmed.1000107)
Supplement: Table S1 — Antibodies and their source used in immunohistologic analysis. (0.06 MB DOC) [file pmed.1000107.s005.doc]

**Table S1: Antibodies and Their Source Used in Immunohistologic Analysis.**

| **Specificity** | **Mab** | **Source** |
| --- | --- | --- |
| human IgG | mouse monoclonal IgG1 clone HP6043 | Hybridoma Research Laboratory, Baltimore, MD |
| human IgA | mouse monoclonal IgG1 kappa clone G20-359 | BD Biosciences, San Jose, CA |
| human IgM | mouse monoclonal IgG1 clone HP6083 | Hybridoma Research Laboratory, Baltimore, MD |
| human kappa | rabbit polyclonal | Dako, Carpinteria, CA |
| human lambda | rabbit polyclonal | Dako, Carpinteria, CA |
| human CD3 | rabbit polyclonal | Dako, Carpinteria, CA |
| human CD68 | mouse monoclonal clone KP1 | Dako, Carpinteria, CA |
| human CD4 | rabbit polyclonal catalogue# sc-7219 | Santa Cruz Biotechnology, Santa Cruz, CA |
| human CD8 | mouse monoclonal clone C8/144B | Dako, Carpinteria, CA |
| human CD20 | mouse monoclonal clone L26 | Dako, Carpinteria, CA |
| human CD11c | mouse monoclonal clone 5D11 | Leica, Bannockburn, IL |
| human Langerin | mouse monoclonal clone 12D6 | Leica, Bannockburn, IL |
| TUNEL | Apop Tag Plus Peroxidase In Situ Apoptosis Kit Catalogue# S7101 | Millipore, Billerica, MA |
| human CD83 | mouse monoclonal IgG1 kappa clone HB15e | BD Biosciences, San Jose, CA |
| human CD205 | mouse monoclonal IgG2b kappa clone MG38 | BD Biosciences, San Jose, CA |
| human CD16 | mouse monoclonal IgG1 kappa clone 3G8 | BD Biosciences, San Jose, CA |
| human CD16 | mouse monoclonal IgG2a clone 2H7 | Research Diagnostics Inc/Fitzgerald Industries, Concord, MA |
| human Ki-67 | mouse monoclonal IgG1 clone MM1 | Vector Laboratories, Burlingame, CA |
| human CD56 | mouse monoclonal IgG1 kappa clone 123-C3 | Dako, Carpinteria, CA |
| human CD57 | mouse monoclonal IgM kappa clone NK-1 | Cell Marque, Rocklin, CA |
| human CD21 | mouse monoclonal IgG1 1F8 | Dako, Carpinteria, CA |
| human CD35 | mouse monoclonal IgG1 BER-MAC | Dako, Carpinteria, CA |
| human nerve growth factor  receptor, p75 | mouse monoclonal IgG1 NGFR5 | Dako, Carpinteria, CA |
