# Supplementary material for: Polyclonal B Cell Differentiation and Loss of Gastrointestinal Tract Germinal Centers in the Earliest Stages of HIV-1 Infection
Source: PLoS Med. 2009 Jul 7;6(7):e1000107. doi: 10.1371/journal.pmed.1000107 (PMC2702159; doi:10.1371/journal.pmed.1000107)
Supplement: Table S2 — Terminal ileal biopsy histologic data. (0.1 MB DOC) [file pmed.1000107.s006.doc]

**Table S2: Terminal Ileal Biopsy Histologic Data.**

| Study Subjects | CD3 | CD4 | CD8 | CD11c | CD68 | IgM | IgA | IgG | κ LC | λ LC | Langerin | CD20 | κ / λ |
| --- | --- | --- | --- | --- | --- | --- | --- | --- | --- | --- | --- | --- | --- |
|  | cells / mm^3^ | | | | | | | | | | | |  |
| AHI off ART |  |  |  |  |  |  |  |  |  |  |  |  |  |
| 017-9 | 502402 | 52557 | 278610 | 61034 | 275784 | 20910 | 74597 | 57220 | 67816 | 41443 | 14693 | 2261 | 1.636 |
| 003-7 | 141283 | N/A | 67251 | N/A | 241311 | 35038 | 233399 | 156541 | 266742 | 158237 | N/A | N/A | 1.686 |
| 019-2 | 560045 | 105491 | 369031 | 98898 | 210794 | 32212 | 79118 | 134219 | 158237 | 112461 | 17519 | 86183 | 1.407 |
| 011-6 | 206838 | 32778 | 127155 | 13563 | 33908 | 27691 | 127155 | 53687 | 105114 | 61599 | 5651 | 19780 | 1.706 |
| 020-4 | 434586 | 19780 | 321560 | 34473 | 134501 | 10172 | 84770 | 18649 | 101159 | 41255 | 1695 | 23170 | 2.452 |
| 023-3 | 184233 | 19780 | 101724 | 28257 | 67251 | 10172 | 58209 | 45211 | 54253 | 42385 | 7912 | 27126 | 1.280 |
| **mean** | 338231 | 46077 | 210888 | 47245 | 160592 | 22699 | 109541 | 77588 | 125553 | 76230 | 9494 | 31704 | 1.695 |
|  |  |  |  |  |  |  |  |  |  |  |  |  |  |
| AHI on ART |  |  |  |  |  |  |  |  |  |  |  |  |  |
| 008-6 | 50297 | 116982 | 74032 | 22040 | 123764 | 4521 | 45776 | 48601 | 53122 | 55383 | 3391 | 7347 | 0.959 |
| 010-1 | 163888 | 11303 | 128850 | 40124 | 68381 | 13563 | 72337 | 38994 | 100593 | 65555 | 8477 | 45211 | 1.534 |
| 018-4 | 211924 | 48601 | 232834 | 5651 | 145239 | 20910 | 92682 | 60469 | 94377 | 49732 | 0 | 46341 | 1.898 |
| 004-5 | 153716 | N/A | 106245 | N/A | 127155 | 22040 | 88160 | 87030 | 101724 | 77988 | N/A | N/A | 1.304 |
| 015-5 | 163323 | 21475 | 308562 | 3391 | 214750 | 24301 | 58209 | 76293 | 98898 | 87595 | 27126 | 9607 | 1.129 |
| 016-8 | 136762 | 17519 | 188189 | 34473 | 197231 | 6216 | 40124 | 34473 | 44080 | 46906 | 10737 | 5651 | 0.940 |
| 016-2 | 196666 | 10172 | 220966 | 51427 | 168974 | 5086 | 3532 | 4521 | 9607 | 3956 | 7347 | 16954 | 2.429 |
| 025-7 | 161628 | 67816 | 157672 | 43515 | 97768 | 31647 | 321560 | 59339 | 284261 | 110201 | 13563 | 55948 | 2.579 |
| **mean** | 154775 | 41981 | 177169 | 28660 | 142908 | 16036 | 90297 | 51215 | 98333 | 62164 | 10092 | 26723 | 1.597 |
| **overall AHI mean** | 233399 | 43688 | 191620 | 36404 | 150486 | 18891 | 98545 | 62518 | 109999 | 68193 | 9843 | 28798 | 1.638 |
|  |  |  |  |  |  |  |  |  |  |  |  |  |  |
| control tissues |  |  |  |  |  |  |  |  |  |  |  |  |  |
| C07 8036 | 126589 | 33343 | 71772 | 25431 | 128285 | 19214 | 118678 | 77988 | 100593 | 120373 | 9607 | 32212 | 0.836 |
| C04 7718 | 102289 | 44080 | 69511 | 19780 | 116982 | 23170 | 105680 | 102289 | 99463 | 87595 | 14128 | 33908 | 1.135 |
| C05 7719 | 116982 | 116982 | 133936 | 11868 | 129980 | 63295 | 202317 | 81944 | 174060 | 131676 | 14128 | 5086 | 1.322 |
| C06-7789 | 58209 | 79118 | 135632 | 36734 | 198361 | 8477 | 41255 | 20910 | 42385 | 29952 | 6216 | 2826 | 1.415 |
| C03 7676 | 50862 | 49449 | 65555 | 6782 | 93812 | 14693 | 66686 | 16954 | 32212 | 37299 | 12998 | N/A | 0.864 |
| C08 8045 | 159932 | 61034 | 68381 | 23170 | 167844 | 27126 | 81944 | 48036 | 63295 | 45211 | 7912 | 2261 | 1.400 |
| **mean** | 102477 | 64001 | 90798 | 20627 | 139211 | 25996 | 102760 | 58020 | 85335 | 75351 | 10832 | 15259 | 1.162 |

Data are represented as cells per mm^3^ in terminal ileum lamina propria as determined by the equation:

| Cells/mm^3^ = | average number of cells |
| --- | --- |
|  | 0.005 mm thickness of the tissue X the area of the 40X field (0.0719 mm^2^). |

N/A = not available.
