# Supplementary material for: Polyclonal B Cell Differentiation and Loss of Gastrointestinal Tract Germinal Centers in the Earliest Stages of HIV-1 Infection
Source: PLoS Med. 2009 Jul 7;6(7):e1000107. doi: 10.1371/journal.pmed.1000107 (PMC2702159; doi:10.1371/journal.pmed.1000107)
Supplement: Table S3 — Total B cell populations in blood in acute HIV-1 infection. (0.04 MB DOC) [file pmed.1000107.s007.doc]

**Table S3: Total B Cell Populations in Blood in Acute HIV-1 Infection.**

|  | **Blood** |
| --- | --- |
| **AHI not on ART**  % B  Absolute Number  Of B Cell/µL | 4.0% ± 0.8%  195 ± 41 |
| **AHI on ART**  % B  Absolute Number  Of B Cell/µL | 5.5% ± 2.1%  326 ± 119 |
| **Uninfected**  % B  Absolute Number  Of B Cell/µL | 3.7% ± 0.4%  227 ± 28 |
