# Supplementary material for: Polyclonal B Cell Differentiation and Loss of Gastrointestinal Tract Germinal Centers in the Earliest Stages of HIV-1 Infection
Source: PLoS Med. 2009 Jul 7;6(7):e1000107. doi: 10.1371/journal.pmed.1000107 (PMC2702159; doi:10.1371/journal.pmed.1000107)
Supplement: Text S1 — Additional methods. (0.04 MB DOC) [file pmed.1000107.s008.doc]

**Supporting Online Material**

**Additional Methods**

For all B cell analyses B cells were gated using both positive and negative criteria prior to analysis. Following singlet gating in both forward and side scatter B cells were selected as CD19^+^ and CD3^−^ CD14^−^ CD16^−^ CD235a^−^ as shown in the left panel. Total B cells were then divided into six groups based on presence or absence of surface IgD expression and the level of CD38 expression [1, 2]. B cell populations present in each group were then further analyzed for the expression of CD27. Naïve B cells were grouped as all surface IgD^+^ cells that were also CD27^−^. Memory B and plasma cells were grouped as all surface IgD^−^ cells that were also CD27^+^; this analysis omitted the population of memory B cells that were surface IgD^+^ IgM^+^ CD27^+^, however in separate analysis this population was found to be similar across all subjects and inclusion of this population did not alter the results of the analyses. The memory B and plasma cell group was further divided into memory B cells (defined as surface IgD^−^ CD38^−/+^ CD27^+^) and as plasma cells / plasmablasts (defined as surface IgD^−^ CD38^hi^ CD27^+^) [1, 2].

References

1. Bohnhorst JØ, Bjørgan MB, Thoen JE, Natvig JB, Thompson KM (2001) Bm1-

Bm5 classification of peripheral blood B cells reveals circulating germinal

center founder cells in healthy individuals and disturbance in the B cell

subpopulations in patients with primary Sjögren's syndrome. J Immunol 167:

3610-3618.

2. Levesque MC, St Clair EW (2008) B cell-directed therapies for autoimmune

disease and correlates of disease response and relapse. J Allergy Clin

Immunol 121: 13-21; quiz 22-13.
